# Stress Inducible Overexpression of *AtHDG11* Leads to Improved Drought and Salt Stress Tolerance in Peanut (*Arachis hypogaea* L.)

**DOI:** 10.3389/fchem.2018.00034

**Published:** 2018-03-02

**Authors:** Jayanna N. Banavath, Thammineni Chakradhar, Varakumar Pandit, Sravani Konduru, Krishna K. Guduru, Chandra S. Akila, Sudhakar Podha, Chandra O. R. Puli

**Affiliations:** ^1^Plant Molecular Biology Laboratory, Department of Botany, Yogi Vemana University, Kadapa, India; ^2^International Crops Research Institute for Semi-Arid Tropics, Patancheru, India; ^3^Molecular Genetics and Functional Genomics Laboratory, Department of Biotechnology, Yogi Vemana University, Kadapa, India; ^4^Department of Biotechnology, Acharya Nagarjuna University, Guntur, India

**Keywords:** peanut, *AtHDG11* (*Arabidopsis Homeodomain globarous11*), drought stress, high-salinity stress, water use efficiency, yield potential

## Abstract

Peanut is an important oilseed and food legume cultivated as a rain-fed crop in semi-arid tropics. Drought and high salinity are the major abiotic stresses limiting the peanut productivity in this region. Development of drought and salt tolerant peanut varieties with improved yield potential using biotechnological approach is highly desirable to improve the peanut productivity in marginal geographies. As abiotic stress tolerance and yield represent complex traits, engineering of regulatory genes to produce abiotic stress-resilient transgenic crops appears to be a viable approach. In the present study, we developed transgenic peanut plants expressing an *Arabidopsis* homeodomain-leucine zipper transcription factor (*AtHDG11*) under stress inducible *rd29A* promoter. A stress-inducible expression of *AtHDG11* in three independent homozygous transgenic peanut lines resulted in improved drought and salt tolerance through up-regulation of known stress responsive genes (LEA, HSP70, Cu/Zn SOD, APX, P5CS, NCED1, RRS5, ERF1, NAC4, MIPS, Aquaporin, TIP, ELIP) in the stress gene network, antioxidative enzymes, free proline along with improved water use efficiency traits such as longer root system, reduced stomatal density, higher chlorophyll content, increased specific leaf area, improved photosynthetic rates, and increased intrinsic instantaneous WUE. Transgenic peanut plants displayed high yield compared to non-transgenic plants under both drought and salt stress conditions. Holistically, our study demonstrates the potentiality of stress-induced expression of *AtHDG11* to improve the drought, salt tolerance in peanut.

## Introduction

Peanut or groundnut (*Arachis hypogaea* L.) is globally important legume crop belonging to the Fabaceae family. Grown for its oil content, feed for both human, and livestock, it forms a major revenue source for marginal farmers as well as for the commercial producers. About 60% of the world peanut production comes from the semi-arid tropics (SAT) such as Africa, Asia, North, and South America, where extremes of temperature, drought, and soil salinity are predominant (Mace et al., 2006; Cuc et al., 2008). Such unfavorable environmental conditions severely affect the plant growth and productivity. Drought and salt stress are the serious constraints affecting both productivity and quality of peanut in SAT regions (Wright and Nageswara Rao, 1994; Reddy and Anbumozhi, 2003; Krishna et al., 2015). It is estimated that drought stress alone can cause an annual loss of 6 million tons worth about 520 million USD (Sharma and Lavanya, 2002; ICRISAT, 2006; Bhatnagar-Mathur et al., 2007). Soil salinity in the SAT regions reduces the plants ability to uptake minerals that inhibits growth and development of peanut, which is known as “saline induced water deficit effect” or “osmotic effect” (Singh and Abrol, 1985; Nautiyal et al., 1989; Singh et al., 1989; Janila et al., 1999).

Development of peanut genotypes with improved drought resistance and WUE (water use efficiency) is highly desirable compared with those varieties developed only with drought tolerance, as always there would be a yield advantage in earlier case (Sharma and Lavanya, 2002). WUE and yield are inter-related complex traits influenced by the interaction of a plentitude of genes related to physiological drought-resistance and yield-associated traits (Araus et al., 2008). As a result, simultaneous selection of several traits for WUE, drought resistance, yield along with elimination of undesirable genomic regions that were co-transferred during breeding programs is difficult task and requires tremendous effort and time (Richards, 1996; Yeo, 1998)., Further, transferring desired traits from wild to cultivated *Arachis* species through classical and molecular breeding has been limited owing to ploidy nature and cross-pollination incompatibility barriers (Janila et al., 2013). Marker assisted selection of useful alleles/QTLs could enhance selection power regardless of breeding constraints.

Genetic engineering or transgenic technology is considered as a versatile and alternative technology to overcome the limitations associated with classical and modern breeding technologies. Significant progress has been achieved in unraveling regulatory mechanisms underlying the drought stress responses and a large number of drought tolerant genes have been identified in a model plant *Arabidopsis* as well as in wide range of plant species (Hauser et al., 2011). Using the identified stress-responsive genes with putative regulatory roles (transcription factors) from the model plants to develop stress resilient crops has proven to be an efficient strategy compared to traditional breeding (Molinar, 2012; Basu et al., 2016). The effective use of transcription factors (TFs) for the development of stress-resilient peanut crop have already been witnessed in previous studies such as heterologous overexpression of individual TFs *AtDREB1A* (Bhatnagar-Mathur et al., 2007; Sarkar et al., 2016), *MuNAC4* (Pandurangaiah et al., 2014), and simultaneous expression of AtDREB2A*, AtHB7*, and *AtABF3* (Pruthvi et al., 2014); *Alfin1, PgHSF4*, and *PDH45* (Ramu et al., 2016). Though drought and salt tolerance mechanisms share common genes, few genes were found specifically involve in salt tolerance. The potential source of salt tolerance genes are haplophytes and their heterologous expression pattern with possible mechanism was thoroughly discussed (Mishra and Tanna, 2017). The constitutive expression of *WRKY3* transcription factor has drastically improved salt tolerance in tomato along with moderate tolerance to drought (Hichri et al., 2017). Apart from transcription factor several other genes like *NHX, HKT*, and *P5CS* were proved efficient for salt tolerance in different genetic backgrounds (Mian et al., 2011; Zhang et al., 2015). To date only a small number of genes directly improving plant WUE and the associated drought tolerance were identified such as ABA-responsive barley gene *HVA1* (Sivamani et al., 2000), a putative transcription factor *ASR1* (Jeanneau et al., 2002), a gene associated with stomatal movement *MRP5-1* (Ghasem et al., 2009), a gene encoding an NADP-malic enzyme (Laporte et al., 2002), an AP2/ERF-like transcription factor gene *HRD* (Karaba et al., 2007), and a *DREB1A* TF gene (Bhatnagar-Mathur et al., 2007). Stress inducible expression system was found better when compared to strong constitutive in improving abiotic stress tolerance through genetic engineering approach (Ben-Saad et al., 2015; Tiwari et al., 2016).

In a recent study Yu et al. (2008) identified and ascertained that overexpression of *Arabidopsis HDG11* gene, a HD-START family transcription factor, confers drought resistance in *Arabidopsis* and tobacco by exhibiting drought tolerant traits such as increased root growth, reduced stomatal density. Further over expression of *AtHDG11* under a constitutive promoter has shown to confer salt, drought stress tolerance, and improved yield in tall fescue, sweet potato, rice, cotton, poplar, wheat, and Chinese Kale (Cao et al., 2009; Ruan et al., 2012; Yu et al., 2013, 2015; Li et al., 2016; Zhu et al., 2016). The *HDG11* transcription factor is a developmental regulator gene that has insignificant role in stress response (Yu et al., 2008). Constitutive overexpression of *HDG11* in transgenic plants accumulates high amount of *HDG11* protein in the nucleus and regulates the expression of several other stress induced genes which are having HD binding sites in their promoters (Yu et al., 2008). However, no experimental data is available on the performance of *AtHDG11* under abiotic stress inducible promoter.

In the present study, we describe the successful transformation and heterologous overexpression of *AtHDG11* gene under the control of a stress inducible *Atrd29A* promoter in a popular peanut variety JL-24 and discuss the performance under drought and salt stress conditions.

## Materials and methods

### Plant material and cloning of *AtHDG11* gene

The seeds of *Arabidopsis thalina* ecotype columbia SALK T-DNA insertion mutant line (SALK_003819) were procured from Arabidopsis Biological Resource Centre, Japan (Yu et al., 2008). The seeds were surface sterilized with 50% liquid bleach containing of 0.05% Tween 20 and sown in a mixture of vermiculite: perlite: peat moss (2:2:1). Pots were kept in the dark for 3 days, at 4°C for stratification and subsequently transferred to normal growth conditions. Thereafter, plants were grown at 22°C under long-day conditions (16-h-light/8-h-dark). Leaves were collected from 30 days old plant and total RNA was isolated using “Nucleospin RNAII kit” (Macherey Nagel). cDNA was prepared using oligo-dT primer, following the procedure described by “Revert Aid premium first strand cDNA synthesis kit” (Life Technologies). For amplification of *HDG11* gene, 1 μL of the reverse transcription reaction was used with Platinum Pfx polymerase (Invitrogen) according to the manufacturer's instructions. The 2.1 kb cDNA fragment of *AtHDG11* (*At1g73360*) was amplified from cDNA using gene-specific primers. The amplified cDNA fragment was column purified (Qiagen) and cloned into *pTZ57R/T* (MBIFermentas) vector following manufacturer's protocol.

### Construction of plant transformation vector with *AtHDG11*

The 2.1 kb of *AtHDG11* coding region was released from *pTZ57R/T* vector using *XhoI* and *NotI* restriction enzymes (NEB) and ligated into *pGreen0029-*binary vector between stress inducible promoter *Atrd29A-*and-*polyA* terminator. The resulting recombinant vector *Atrd29::AtHDG11:PolyA* was mobilized along with the helper vector *pSoup* into *EHA105* strain of *Agrobacterium tumefaciens* by electroporation (Eppendorf) method. Recombinant colony of *A. tumefaciens* strain carrying the construct was selected using colony PCR method. Selected colony was cultured in YEP broth (50 μg/mL) and rifampicin (10 μg/mL) and allowed to grow at 28°C on a shaker with 180 rpm. When the OD (A_600_) reached 0.4–0.5, the bacterial culture was removed and centrifuged at 5000 rpm for 10 min at 4°C, the pellet was dissolved in 50 mL of half strength MS media with 100 μM of acetosyringone and the suspension was used for co-cultivation. *PGreen0029-binary* vector without any transgene served as an empty vector control.

### Plant transformation

Peanut (*Arachis hypogaea* L. *cv*. JL24) seeds were obtained from Regional Agriculture Research Station, Kadiri, Andhra Pradesh, India. Mature seeds were surface sterilized with tween-20 for 5–8 min then with 0.1% (w/v) aqueous mercuric chloride (HgCl_2_) for 7 min followed by 5–6 rinses with sterile double distilled water. Blot dried seeds were inoculated on half strength MS media for obtaining aseptic seedlings. Cotyledonary node (CN) explants were prepared from 6 days old aseptic seedlings as explained by Beena et al. (2005). The CN explants were independently immersed in the bacterial suspensions carrying the construct and empty vector for 5 min and blot dried by placing between the sterile filter paper to remove excess of bacterial suspension and explants were transferred to the regeneration media. Regeneration protocol was followed as discussed by Anuradha et al. (2006).

### Analysis of transformants

#### Kanamycin analysis

All the T_1_ and T_2_ generation seeds were collected from transgenic plants, surface sterilized with 1% (w/v) bavistin and allowed to grow on half strength MS media consisting of 200 mg/L kanamycin, along with the non-transgenic seeds for 10–15 days at 28°C. Healthy plants with green leaves and having good root and shoot ratio were selected as positives.

#### Molecular analysis of putative transgenic plants for stable transgene integration and segregation

Genomic DNA was isolated from T_1_, T_2_, and T_3_ putative transgenic and non-transgenic plants using CTAB method (Doyle and Doyle, 1987). PCR analysis was carried out with different combinations of gene, promoter, and *nptII* specific primers for detection of cloned components. PCR amplification was carried out in a 25 μL reactions containing 100 ng of genomic DNA as a template. The PCR was initiated by a hot start at 94°C for 5 min, 30 cycles of 94°C for 1 min, 55°C for 1 min, and 72°C for 2 min. The amplicons were resolved on 0.8% agarose gel electrophoresis.

Southern analysis was carried out using 20 μg of purified genomic DNA from selected T_2_ PCR confirmed transgenics along with non-transgenic plants. DNA was digested with *XhoI* enzyme, electrophoresed, and blotted onto a positively charged nylon membrane (Hybond-N^+^). Radiolabeled PCR product of *AtHDG11* gene was used as a probe. Hybridization and washing were carried out at 58°C as described by Sambrook et al. (1989).

#### Screening for drought and salt stress tolerance by outdoor lysimetric dry-down experiment

The abiotic stress response in selected T_3_ transgenic plants along with NT plants were carried out under field conditions using the lysimetric dry-down conditions as described by Jagana et al. (2012). Selected peanut transgenic lines were grown in the PVC lysimeters (120 × 20 cm) consisting of 50 kg soil. Plants were watered regularly for 1 month to gain full field capacity. Drought stress was imposed in 1 month old plants by withholding the water and salt stress was imposed by adding 250 mM salt (NaCl) solution to the cylinders. Drought and salt stress were continued for 30 days. Field capacity of drought stressed plants was regularly monitored by weighing the plants using an electrical balance connected with a pulley. Leaf samples were collected with 10 days interval (10, 20 and 30 days) for drought and 5 days interval (5, 10, and 15 days) for salt stress; after stress imposition. Thirty days after imposition of drought stress, NT, and transgenic plants were rehydrated by adding the water to their field capacity and allowed for rehydration. Rehydration capacities of non-transgenic and transgenic plants were measured based on their recovery from drought stress and samples were collected after 5 days of recovery.

### Quantitative real time PCR (qRT-PCR)

The total RNA for qRT-PCR analysis was isolated using Nucleospin RNAII kit (Macherey Nagel) from leaf tissues of transgenic (T_3_ generation) and non-transgenic (NT) plants. After DNAse I (Fermentas) treatment, RNA was used for cDNA synthesis using RevertAid™ premium First strand cDNA synthesis Kit (Fermentas). Real-time PCR was carried out using FastStart universal SYBR green master mix (Roche Applied Science) as instructed in the manual with *AtHDG11* gene specific primers F-5′-ATC CAA CAA CAC ACG CTC AA-3′ and R-5′-AGA AGG ATC TTC ACC GCT CA−3′. ADH3 (EG529529) was used as an internal control with F-5′*-*GCTTCAAGAGCAGGTCACAAGT-3′ and R-5′-GAGACATCCTCCTTCGTGCATA-3′ as primers. Amplicons were analyzed on Roche Light Cycler 480 II Real-Time PCR System according to the manufacturer's instructions. The average values of three biological samples were used to calculate the relative fold expression. The relative fold in expression levels were calculated using qbase^+^Software (Hellemans et al., 2007) by normalizing with Actin reference gene (Condori et al., 2011).

Known stress responsive genes (Supplementary Table 1) expression was measured in homozygous peanut transgenic lines under drought and salt stress conditions using qRT-PCR. RNA samples were isolated from the NT and transgenic lines on 10th day after drought stress imposition and day 5th after salt stress and their respective control well-watered plants grown in the lysimetric experiment. Total RNA isolation, cDNA synthesis and qRT-PCR were performed as mentioned in the previous section.

### Chlorophyll estimation

Chlorophyll content of non-transgenic type and transgenic plants grown in the lysimetric experiment under well-water, drought, and salt stress conditions was measured using SPAD chlorophyll meter (SPAD-502, Konica Minolta, Japan) according to Rao et al. (2001) and Qin et al. (2013). SPAD chlorophyll meter readings () were taken from the fully expanded third leaf of non-transgenic and transgenic lines at a 10 days interval (10, 20, and 30 days) for drought and 5 days interval (5, 10, and 15 days) for salt stress.

### Specific leaf area (SLA)

Specific Leaf Area of NT and transgenic plants grown in the lysimetric experiment under well-water, drought, and salt stress conditions was measured using a leaf area meter (LI-3000C LI-COR Biosciences) according to Sebahattin and Necdet (2007). SLA readings were taken from the fully expanded third leaf of NT and transgenic lines at 10 days interval (10, 20, and 30) for drought and 5 days interval (5, 10, and 15) for salt stress. To determine the leaf dry weight, leaf samples were oven dried at 80°C for 48 h. SLA was calculated using the following formula.

$$\begin{array}{l} \text{SLA}=\frac{\text{Leaf area }\left(\text{c}{\text{m}}^{2}\right)}{\text{Leaf dry weight }\left(\text{g}\right)} \end{array}$$

### Relative water content (RWC)

Relative Water Content in the leaves of NT and transgenic plants was measured according to the (Gonzales and Gonzales-Vilar, 2001). Four leaflets from the top leaves of the main stem were used for the measurement of RWC. Initially, fresh weights (FW) of the harvested leaves were recorded within 15 min, then leaf samples were floated on 20 mL distilled water for 5 h to attain full turgidity and turgid weight (TW) was recorded. Then leaflets were oven dried at 80°C for 48 h and dry weight (DW) of the leaflets were recorded. The leaf relative water content was calculated using the following formula.

$$\begin{array}{l} \text{RWC}\left(\%\right)=\frac{\text{FW}-\text{DW}}{\text{TW}-\text{DW}}\times 100 \end{array}$$

### Determination of stomatal size and density

Stomata size [Stomatal Length (STL LEN) & Stomatal width (STL WID)] and Stomatal density (STL DEN) of transgenic and NT plants were determined from the fully expanded third leaf of NT and transgenic lines after 10 days of drought and 5 days after salt stress using leaf surface imprint method (Yu et al., 2008). The leaf imprints were collected and placed on glass slide and observed under Olympus microscope (BX51, *Olympus*). Stomatal density and size were determined using OLYMPUS Image Analysis software.

### Determination of photosynthetic rate, transpiration rate, and water use efficiency

Gas exchange parameters such as photosynthetic rate (P) (μmolm^−2^ s^−1^) and Transpiration rate (T) (mmol^−2^ s^−1^) were measured in NT and transgenic plants using a portable photosynthetic system Li-6400 (LI-COR, USA) according to (Babitha et al., 2015). Three measurements were taken from the fully expanded third leaf of NT and transgenic lines at 10 days interval (10, 20, and 30) for drought and 5 days interval (5, 10, and 15) for salt stress in the morning (9–11 a.m.). Water Use Efficiency (WUE) was derived from P/T ratio.

### Measuring root parameters

After the stress period, plants were harvested carefully without disturbing the root system from PVC lysimeter tubes. Shoot and root lengths were measured in centimeters (cm) using standard scale. Fresh weights of root and shoots, number of lateral roots and, nodules were also recorded. Further, shoots and roots were oven dried at 60°C for 48 h and weights were recorded for transgenic and NT plants.

**Yield**: Numbers of pods and seeds per plant were counted after harvesting the plants. Dry weight was taken after complete drying of the pods in an oven at 60°C and expressed in terms of grams per plant.

**Drought Tolerance Index**: Drought tolerance index (DTI) of NT and transgenic plants grown in the lysimetric experiment under well-water and drought stress conditions was measured based on the pod yield according to Nautiyal et al. (2002), using following formula.

$$\begin{array}{l} \text{DTI}=\frac{\text{Pod yield under stressed conditions}}{\text{Pod yield under non–stressed conditions}} \end{array}$$

**Harvest index (HI)**: Harvest index was calculated using the following formula as described by Painawadee et al. (2009).

$$\begin{array}{l} \text{HarvestIndex}\left(\text{HI}\right)=\frac{\text{Pod yield}}{\text{Pod yield}+\text{shoot and root dry weight}} \end{array}$$

### Measurement of free proline and antioxidative enzymes

Estimation of proline in the leaves of NT and transgenic plants grown in the lysimetric experiment under well-water, drought, and salt stress conditions was measured according to Bates et al. (1973). Antioxidative enzymes such as superoxide dismutase (SOD), catalase (CAT), and ascorbate peroxidase (APX) were quantified in the NT and transgenic plants grown in the lysimetric experiment under well-water, drought, and salt stress conditions according the method described by Elavarthi and Martin (2010).

### Lipid peroxidation (MDA) and electrolyte leakage

Leaf samples from the stressed and control samples from transgenic and NT plants were used for the measurement of malondialdehyde (MDA) concentration (a product of lipid peroxidation) as described by Quan et al. (2004). Leaf discs were punched from the stress treated and control samples and membrane electrolyte leakage was assayed as described by Shou et al. (2004).

### Statistical analysis

Chi square (X^2^), probability (*P*) values, correlation analysis and ANOVA (Duncan method) were done by using Software IBM SPSS Statistics v. 2.0. Graph data represents mean± S.E (*n* = 3). Cluster analyses were performed and heat maps were generated to group the physiological and biochemical traits based on the degree of correlation with R Statistical Environment (R Core Team, 2012).

## Results

### Developing transgenic construct

Full length cDNA clone of *AtHDG11* was successfully isolated from *A. thalina* mutant line SALK_003819 and cloned initially into *pTZ57R/T* vector using *XhoI* and *NotI* restriction enzymes and later into plant transformation vector *pGreen0029* in between *At rd29A* promoter and *polyA* terminator. Conformation of the individual clones in the construct was done through PCR analysis, restriction digestion, and sequencing of the *HDG11* gene.

### Development of transgenic peanut plants

Five hundred cotyledonary node (CNN) explants in five batches were co-cultivated with *Agrobacterium* strain (EHA105) harboring *AtHDG11* (Supplementary Figure 1A) by exposing the proximal cut ends of explants in the bacterial suspension. Following infiltration, the explants were transferred to shoot induction media (SIM) (Supplementary Figure 1B) supplemented with kanamycin (200 mg/L) monosulphate and cefotaxime (10 μg/mL) as selection agent. The well-differentiated 312 shoot-lets (62.4%) were transferred to shoot elongation medium (SEM) comprising kanamycin (200 mg/L) and cefotaxime (10 μg/mL). Shoot-lets were further sub-cultured on SEM with 2 week growth interval to ensure further selection of transgenics. Well-developed 262 shoot-lets (52.4%) were transferred to root induction medium containing NAA (0.8 mg/L) without antibiotic. Finally 125 plants were recovered (25%) with fully developed root system that were placed in sterile vermiculite containing pots. After hardening for 2 weeks, 62 plant-lets (12.4%), were transferred to earthen-pots containing sterile soil and sand mixture (3:1) and grown under greenhouse conditions. The survived 14 putative transgenic plants (2.8%) were subjected for functional validation using various biochemical and physiological parameters.

### Molecular characterization and transgene inheritance analysis

Integration and segregation analysis of transgene in T_1_& T_2_ generation was carried out using PCR and southern blot analysis. PCR analysis in 14 (T_0_) putative transgenics using different combinations of primers has revealed, eight plants were positive for all components of the transgenic construct (Supplementary Figure 2A and **Figure 2B**). Eight (T_0_) putative transgenic peanut plants were advanced to T_1_ through selfing and the progeny populations (T_2_) were analyzed for transgene integration and stability through PCR and transgene inheritance analysis was carried out in eight populations to know the zygosity nature of transgenic lines. Southern blot analysis carried out in seven independent T_2_ transgenic lines and integration of single copy of transgene has been confirmed in three transgenic lines (Supplementary Figure 2D lane: 1–3) and multiple copies of integration in three lines (Supplementary Figure 2D lane: 5–7), while no transgene integration was found in one line (Supplementary Figure 2D lane: 4). The single copy integrated T_2_ individual transgenic lines were advanced to T_3_ generation by selfing. The χ2 analysis of transgene inheritance was presented in Table 1.

**Table 1:** Segregation analysis of *AtHDG11* gene in self progenies (T1) derived from *AtHDG11* transgenic plants.

**T_1_-Generation**	**Total number of seeds**	**Kan & PCR +Ve**	**Kan & PCR –Ve**	**Ratio**	**Test Ratio**	**X^2^**	***P***
Plant-1	11	8	3	2.6	3:1	0.030	0.862
Plant-2	12	9	3	3	3:1	0.00	1
Plant-3	No seeds	–	–	−	0	0	0
Plant-4	11	8	3	2.6	3:1	0.030	0.862
Plant-5	10	7	3	2.3	3:1	0.133	0.715
Plant-6	6	4	2	2	3:1	0.22	0.637
Plant-7	No seeds	–	–	−	0	0	0
Plant-8	7	5	2	2.5	3:1	0.048	0.827

### Transgenic peanut plants over expressing *HDG11* showed enhanced abiotic stress tolerance

**Figure 1:**
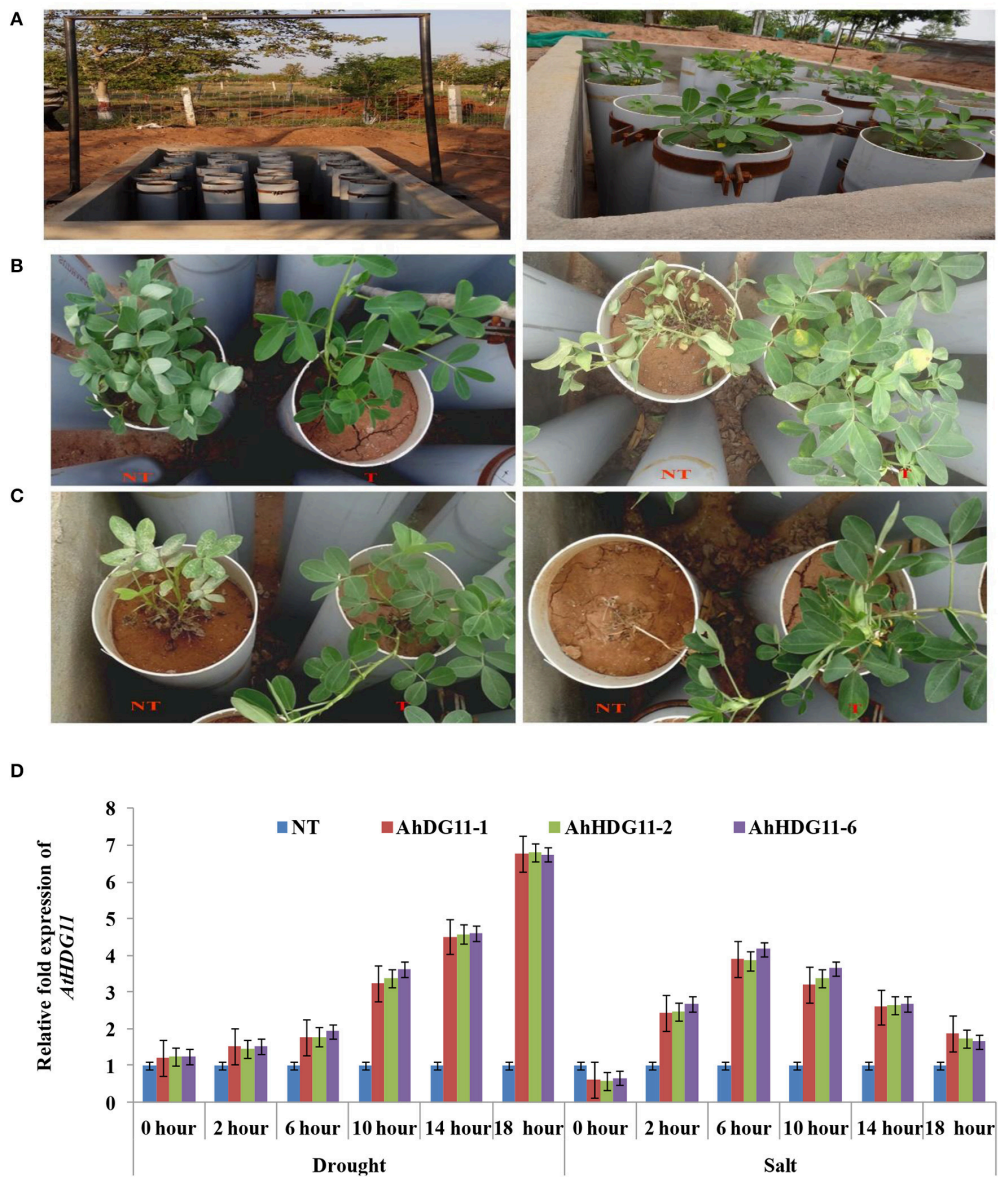
Screening of non-transgenic and transgenic plants for drought and salt tolerance using lysimetric experiment. **(A)** Lysimetric experiment setup. Representative pictures of stress-induced phenotype of non-transgenic (NT) and transgenic (T) lines under **(B)** drought stress,15 days of stress imposition (left panel) and 30 days of stress imposition (right panel) **(C)** salt stress, 7 days of stress imposition (left panel) and 15 days of stress imposition (right panel). **(D)** qRT-PCR analysis of the T_3_ generation peanut transgenic lines for the *AtHDG11* transcript at various time points under induced drought and salt lysimetric experiments to check the stress-inducible expression of transgene *AtHDG11* under *rd29A* promoter.

Progeny plants derived from single copy integrated transgenics were designated as *Ah-HDG11-1, Ah-HDG11-2*, and *Ah-HDG11-6*. These transgenics were characterized for their abiotic stress tolerance in an outdoor lysemetric experiment using various physiological and biochemical parameters (Figure 1A). No significant phenotypic variation was observed between transgenic and non-transgenic (NT) plants under well-watered conditions (data not shown). However, upon imposition of drought stress NT plants exhibited visual wilting symptoms on day 10th, while on the other hand transgenic lines, displayed trivial visual wilting symptoms only on day 30th of post drought stress imposition (Figure 1B). To evaluate the post stress recovery capacity, transgenic lines, and non-transgenic plants were re-watered to their saturated filed capacity and monitored the recovery. All the three independent transgenic lines recovered with a survival rate of 97.8% from drought stress, whereas the NT plants showed a survival rate of only 5%.

**Figure 2:**
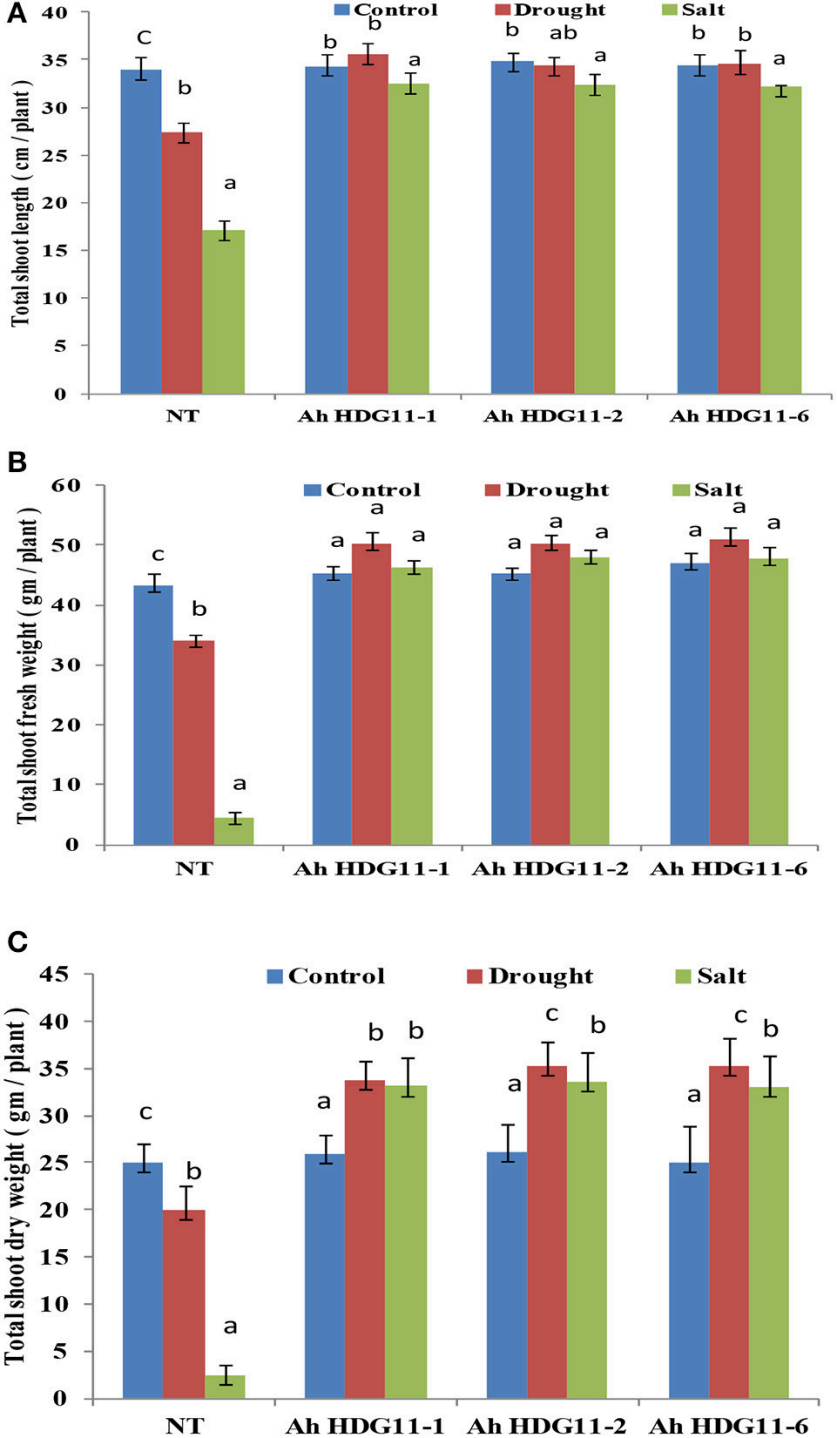
Post-harvest shoot growth and biomass analysis of three transgenic lines *AhHDG11-1, AhHDG11-2, AhHDG11-6* along with the control untransformed parent JL-24 (NT) under control (well-watered), drought and salt stress from the confined outdoor lysimetric experiment. **(A)** Shoot length (cm/plant). **(B)** Shoot fresh weights (gm/plant). **(C)** Shoot dry weights (gm/plant). Alphabets in lowercase indicates the statistical significance in differences between WT and transgenics at *p* < 0.05.

To study the role of *AtHDG11* in regulating the salinity response, the transgenic lines and NT plants were exposed to high salinity stress conditions. On exposure to 250 mM NaCl, NT plants showed visual symptoms of chlorosis, severe growth retardation on 5th day after stress imposition, and eventually died after 15 days (Figure 1C). In contrast, transgenic lines did not exhibit salt-induced stress symptoms even after 15 days of salt stress imposition. Transgenic plants recorded increase in fresh and dry weights of shoot and root compared to NT plants, both under drought and salt stress conditions (Figures 2A–C, **4B,C**). These results indicate that *AtHDG1*1 promotes growth and tolerance under drought and salt stress conditions in transgenic peanut plants.

### Drought and salt stress-inducible expression of transgene *AtHDG11*

To validate the stress inducible nature of *rd29a* promoter, a qRT-PCR experiment was carried out with the samples recovered from lysimetric dry-down stress experiments at different time intervals. As shown in the Figure 1D, a gradual increase in the expression of *At HDG11* transcript was observed in the transgenic lines under drought and salt stress, while no transcripts were detected in the NT. The data clearly indicates the tight regulation of transgene expression during the drought and salt stress conditions.

### Transgenic plants displayed improved yield under drought and salt stress conditions

**Figure 3:**
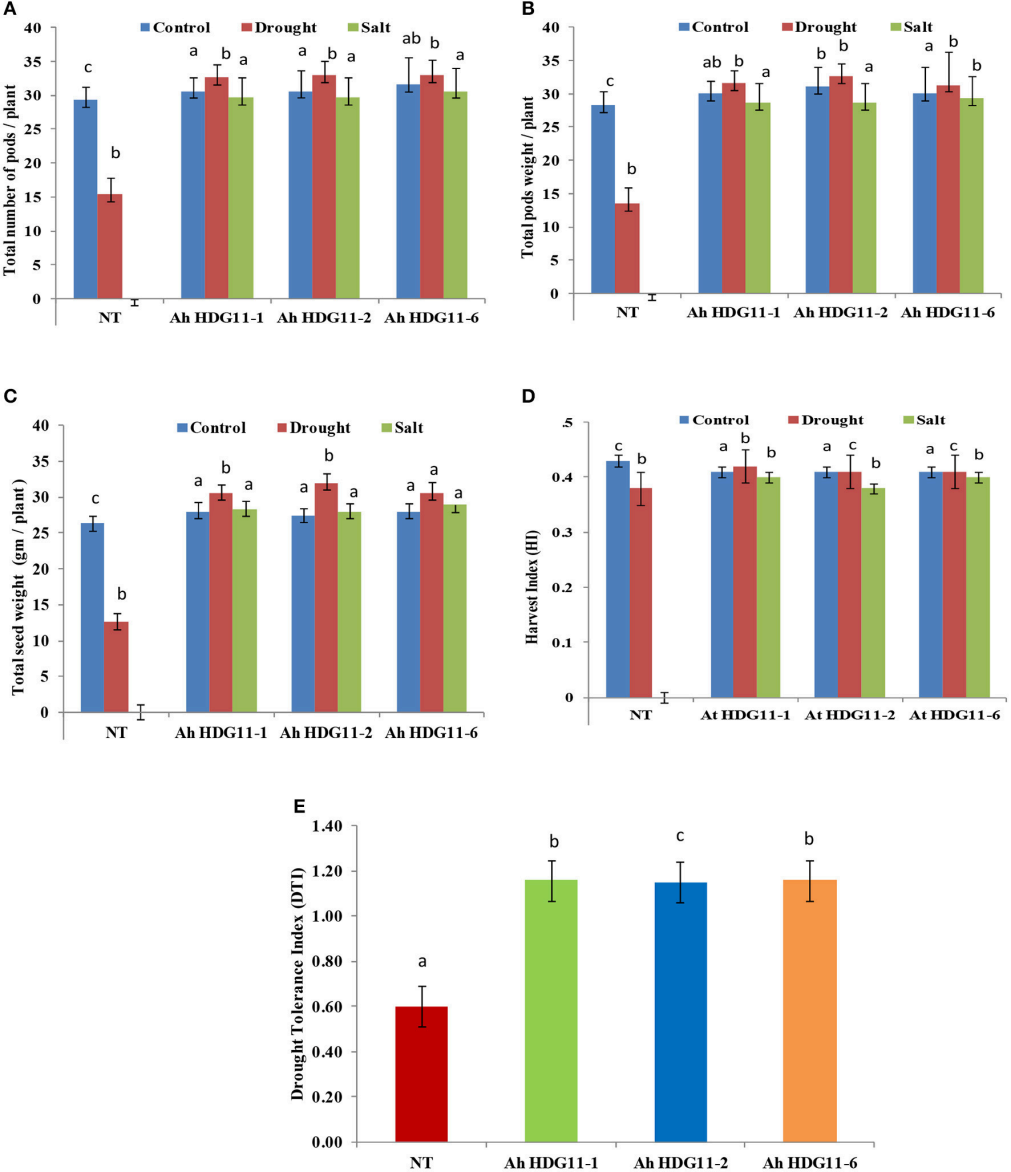
Yield analysis of three transgenic lines *AhHDG11-1, AhHDG11-2, AhHDG11-6* along with the control untransformed parent JL-24 (NT) under control (well-watered), drought, and salt stress from the confined outdoor lysimetric experiment. **(A)** Total number of matured pods/plant. **(B)** Pod weight (gm/plant). **(C)** Seed weight (gm/plant). **(D)** Harvest index. (**E)** Drought Tolerance Index (DTI). Alphabets in lowercase indicates the statistical significance in differences between WT and transgenics at *p* < 0.05.

Yield in terms of pod number, pod, and seed weight were measured in non-transgenic (NT) as well as in transgenic lines of *Ah-HDG11-*1, *Ah-HDG11-2*, and *Ah-HDG11*-6 under well-watered (WW), drought, and salt-stress conditions (Figures 3A–D). Pod yield has drastically reduced (15 pods/plant) in NT plants compared to transgenics (30–32/plant) under drought and high salinity stress (Figure 3A). Considerable increase in the weight of the seed in transgenic lines has been noticed. No significant variation in yield and weight of the seeds was observed among the transgenic lines.

### Transgenic plants exhibited improved water-use efficiency (WUE) traits

**Figure 4:**
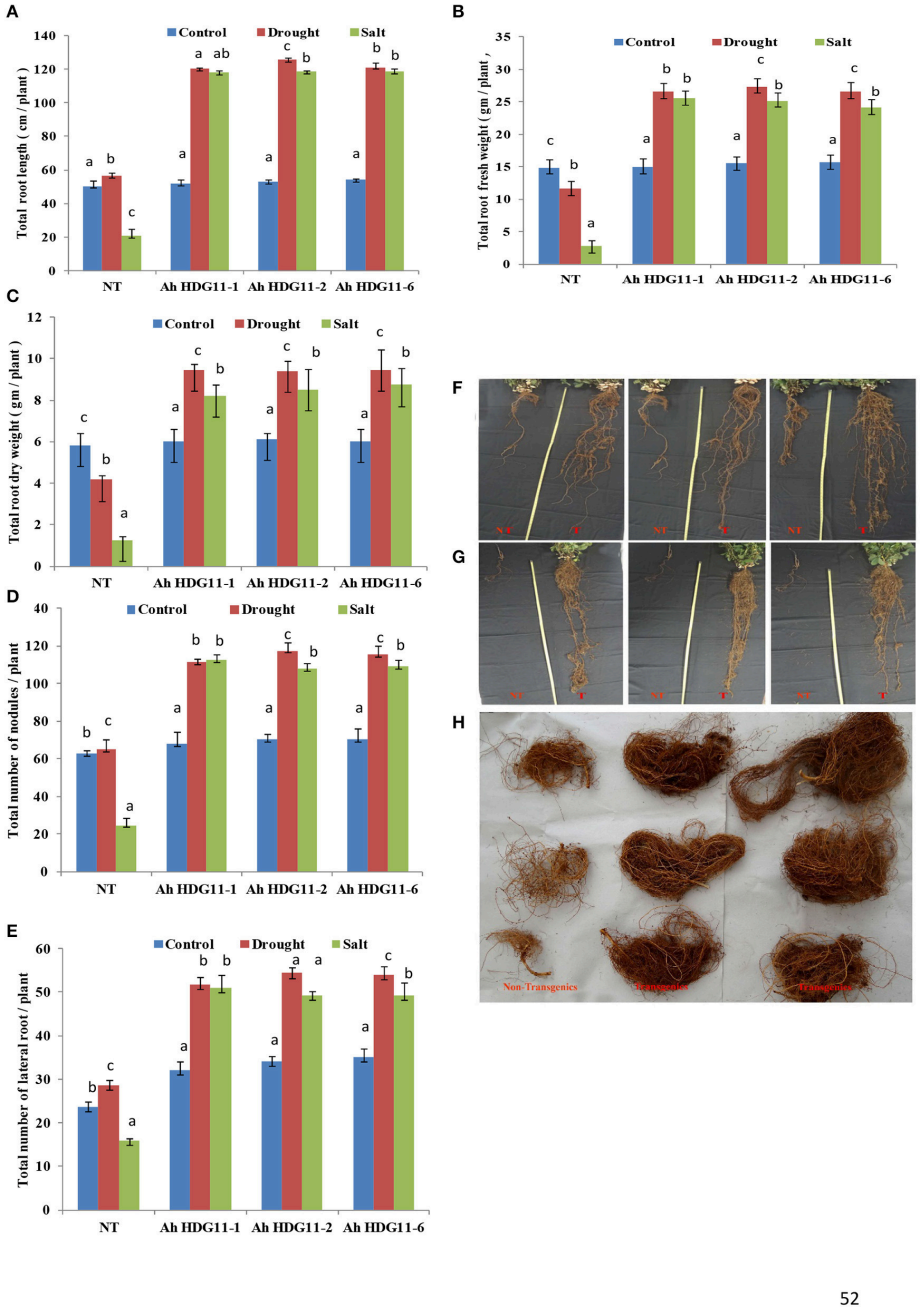
Post-harvest root morphology, growth, and biomass analysis of three transgenic lines *AhHDG11-1, AhHDG11-2* and *AhHDG11-6* along with the control untransformed parent JL-24 (NT) under control (well-watered), drought and salt stress from the confined outdoor lysimetric experiment. **(A)** Root length (cm/plant). **(B)** Root fresh weight (gm/plant). **(C)** Root dry weight (gm/plant). **(D)** Root nodules. **(E)** Lateral roots. **(F)** Phenotype of roots under drought stress. **(G)** Phenotype of roots under salt stress. **(H)** Root mass of Non-transgenic (NT) and transgenic plants (T). Alphabets in lowercase indicates the statistical significance in differences between WT and transgenics at *p* < 0.05.

**Figure 5:**
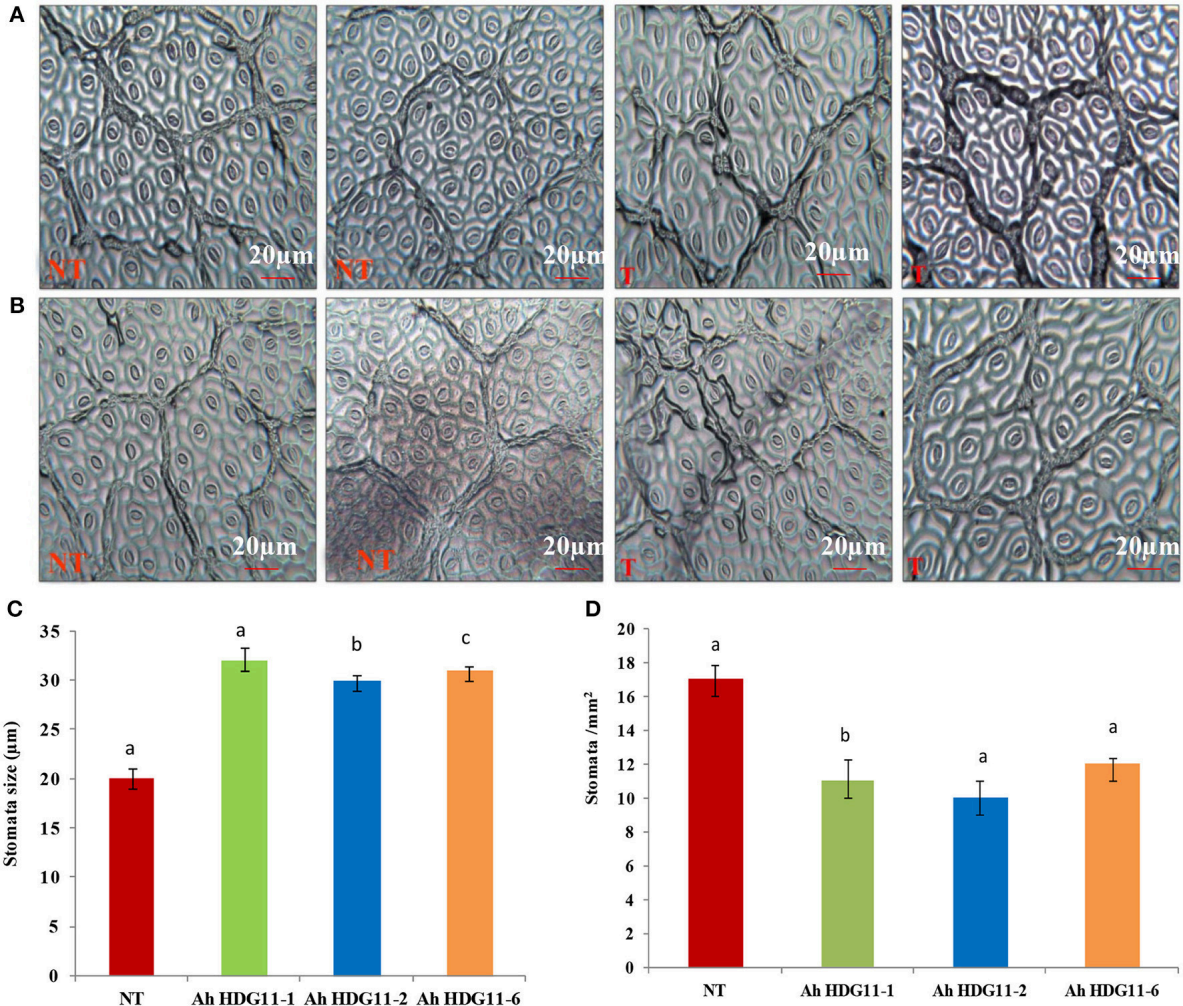
Stomatal analysis of three transgenic lines *AhHDG11-1, AhHDG11-2, AhHDG11-6* along with the control untransformed parent JL-24 (NT) under control (well-watered), drought, and salt stress from the confined outdoor lysimetric experiment. Adaxial epidermal imprint image of **(A)** Drought stressed plants. **(B)** Salt stressed plants. **(C)** Stomatal width (μm). **(D)** Stomatal density (Stomata/mm^2^).

Transgenic peanut lines over expressing *AtHDG11* developed deeper root system with increased number of lateral roots and root nodules (Figures 4A,D,E) both under drought and salt-stress conditions. As shown in the (Figures 4F,G) transgenic lines showed larger root systems (50–75%), root biomass (60–70%) (Figure 4H), and root nodules (55–65%) compared to NT plants. Stoma plays a key role in keeping the oxidative stress at pace in plants by regulating the transpiration. In order to understand the role of stomata in enhanced WUE of transgenic plants, we analyzed the stomatal density in the transgenic and NT plants both under drought and salt stress conditions. The adaxial stomatal density and size of the 3rd fully expanded leaf were determined by leaf surface imprint method. The average stomatal densities of the selected transgenic lines were decreased by 50–60% compared with that of the NT plants (Figures 5A,B,D). However, the stomatal size (length and width) was increased by 20% in the transgenic plants compared to NT (Figure 5C).

**Figure 6:**
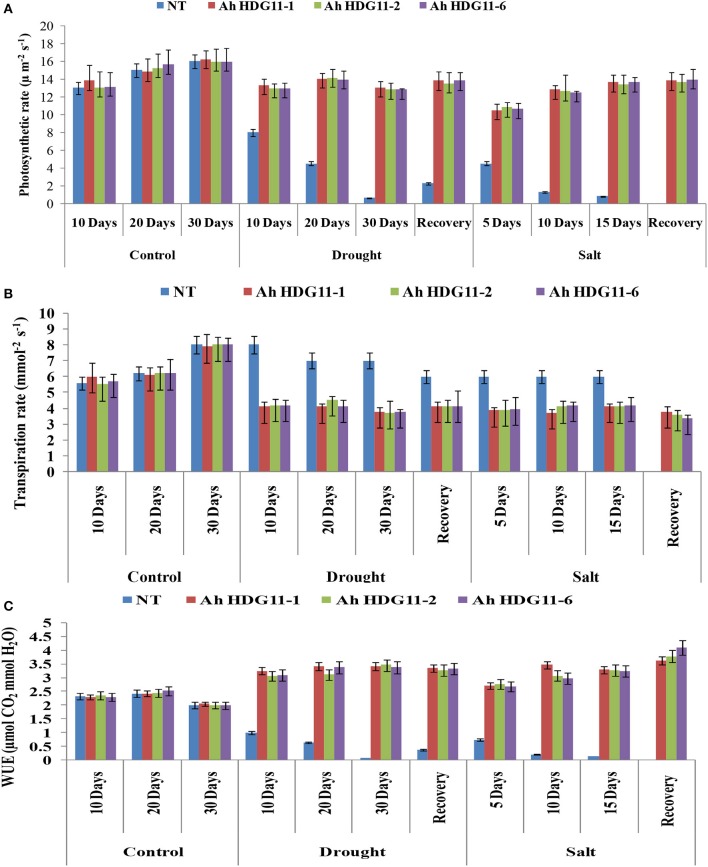
Gas exchange and Water Use Efficiency Analysis of transgenic lines *AhHDG11-1, AhHDG11-2, AhHDG11-6* along with the Non-transgenic (NT) plants. **(A)** Photosynthesis rate **(B)** Transpiration rate **(C)** Water Use Efficiency.

In order to know whether the reduced stomatal density affected the photosynthesis or not, the photosynthesis and transpiration rates of NT and transgenic peanut lines were measured with a 10 days interval (10, 20, and 30th days) after drought stress imposition, 5, 10, and 15th day of salt stress stressed plants and 5 days after recovery from the stress. Under well-water conditions both transgenic and non-transgenic lines recorded similar photosynthetic rates, however, under drought and salt stress conditions photosynthetic rates were drastically reduced in non-transgenic plants. Interestingly, though the number of stomata decreased in transgenic lines, they recorded 30–50% higher photosynthetic rates compared to non-transgenic lines and maintained higher photosynthetic rates even under severe drought and salt stress conditions (Figure 6A). In contrast, transgenic lines under drought and salt stress conditions recorded lower transpiration rates compared to non-transgenic plants (Figure 6B). As a result transgenic plants recorded higher WUE rates under both drought and salt stress conditions (Figure 6C).

**Figure 7:**
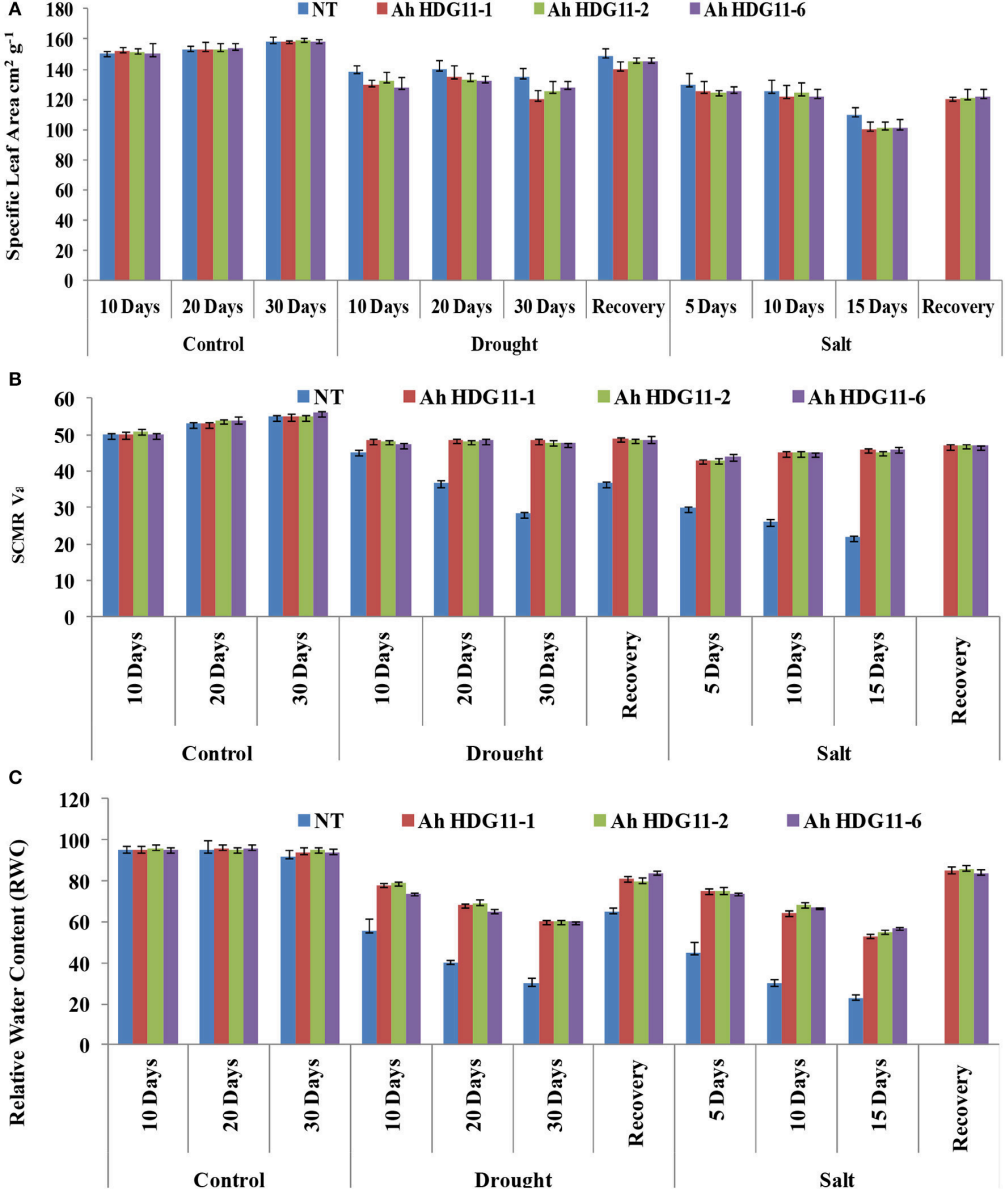
Physiological analysis of transgenic lines *AhHDG11-1, AhHDG11-2, AhHDG11-6* along with the Non-transgenic (NT) plants. **(A)** Specific Leaf Area (SLA) **(B)** Chlorophyll content (SCMR) **(C)** Relative Water Content (RWC).

Traits such as SLA and SCMR were used as indirect surrogates for measuring WUE. The SLA and SCMR readings were measured from drought and salt stress imposed plants for every 5 days interval and 5 days after recovery from the stress. Transgenic lines of *Ah-HDG11-*1, *Ah-HDG11-2*, and *Ah-HDG11*-6 recorded higher SCMR values compared to NT plants during drought and salt stress (Figure 7B). SCMR values increased with duration of both drought and salt stress and highest SCMR values were recorded in transgenic lines of *Ah-HDG11-*1. Relatively, high SCMR values were observed in drought stress recovered transgenic lines of *Ah-HDG11-*1, *Ah-HDG11-2*, and *Ah-HDG11*-6 compared to NT plants under WW conditions. Low SLA values were recorded in transgenic lines of *Ah-HDG11-*1, *Ah-HDG11-2*, and *Ah-HDG11*-6 (Figure 7A) under drought and salt stress conditions compared to their control plants and NT plants. SLA was decreased with increased duration of stress.

To investigate the current water content in the transgenic and non-transgenic plants relative to their maximal water content they can hold at full turgidity under drought and salt stress conditions, relative water content (RWC) was measured. A sharp decline in the RWC was noticed in both non-transgenic as well as in transgenic plants with increase in the severity of drought and salt stress. However, transgenic lines Ah*-AtHDG11*-1, *Ah-AtHDG11*-2, and *Ah-AtHDG11*-6 maintained higher RWC (78% on day 10, 68%on day 20, and 60% on day 30th) of drought stress in their leaves (Figure 7C). Whereas, non-transgenic plant leaves had RWC of (55% on day 10, 40% on day 20, and 30% on day 30th) of drought stress. Up on release of drought stress, fast recovery of RWC observed in transgenic plants (85% RWC) compared to non-transgenic plants (65% RWC) (Figure 7C). Similar pattern was also observed in the salt-stress imposed transgenic and non-transgenic plants (Figure 7C).

The correlation between the traits contributing to WUE in drought and salt stress are listed in Table 2. Root traits (RL, RFW, and RDW), chlorophyll content (in relative SPAD units), RWC, TE, HI, DTI, STL LEN, and STL WID under drought and salt stress, were positively correlated with each other. Apparently, SLA under drought stress negatively correlated with root traits (RL−0.61; RFW−0.63; RDW−0.61), chlorophyll content (−0.64), RWC(−0.61), TE (−0.62), HI (−0.56), DTI (−0.62), STL LEN (−0.69), and STL WID (−0.69), while SLA in drought stress positively correlated with stomatal density (STL DEN 0.64), and negatively correlated with STL DEN (−0.75) in salt stress conditions. Stomatal density in drought and salt stresses negatively correlated with all WUE traits, except with SLA in drought stress (0.64) (Table 2).

**Table 2:** Correlation coefficient (*r*) between various morpho-physiological traits contributing to WUE in peanut *AtHDG11* transgenic lines under drought and salt stress.

	**RL (D)**	**RL (S)**	**RFW (D)**	**RFW (S)**	**RDW (D)**	**RDW (S)**	**SPAD(D)**	**SPAD(S)**	**RWC(D)**	**RWC (S)**	**SLA(D)**	**SLA(S)**	**TE (D)**	**TE (S)**	**HI (D)**	**HI (S)**	**DTI (D)**	**DTI (S)**	**STL DEN (D)**	**STL DEN (S)**	**STL LEN (D)**	**STL LEN (S)**	**STL WID (D)**	**STL WID (S)**	
RL (D)	1																								
RL (S)	0.99^**^	1																							
RFW (D)	0.99^**^	0.99^**^	1																						
RFW (S)	0.99^**^	0.99^**^	0.99^**^	1																					
RDW (D)	0.99^**^	0.99^**^	0.99^**^	0.99^**^	1																				
RDW (S)	0.99^**^	0.99^**^	0.99^**^	0.99^**^	0.99^**^	1																			
SPAD(D)	0.99^**^	0.99^**^	0.99^**^	0.99^**^	0.99^**^	0.99^**^	1																		
SPAD(S)	0.99^**^	0.99^**^	0.99^**^	0.99^**^	0.99^**^	0.99^**^	0.99^**^	1																	
RWC(D)	0.99^**^	0.99^**^	0.99^**^	0.99^**^	0.99^**^	0.99^**^	0.99^**^	0.99^**^	1																
RWC (S)	0.98^*^	0.99^**^	0.99^**^	0.99^**^	0.99^**^	0.99^**^	0.99^**^	0.99^**^	0.99^**^	1															
SLA(D)	−0.61	−0.61	−0.63	−0.63	−0.61	−0.57	−0.64	−0.62	−0.61	−0.53	1														
SLA(S)	0.78	0.78	0.75	0.76	0.78	0.8	0.75	0.76	0.77	0.82	0	1													
TE (D)	0.99^**^	0.99^**^	0.99^**^	0.99^**^	0.99^**^	0.99^**^	0.99^**^	0.99^**^	0.99^**^	0.98^*^	−0.62	0.76	1												
TE (S)	0.99^**^	0.99^**^	0.99^**^	0.99^**^	0.99^**^	0.98^*^	0.99^**^	0.99^**^	0.99^**^	0.97^*^	−0.68	0.72	0.99^**^	1											
HI (D)	0.99^**^	0.99^**^	0.99^**^	0.99^**^	0.99^**^	0.99^**^	0.99^**^	0.99^**^	0.99^**^	0.99^**^	−0.56	0.82	0.99^**^	0.98^*^	1										
HI (S)	0.99^**^	0.99^**^	0.99^**^	0.99^**^	0.99^**^	0.99^**^	0.99^**^	0.99^**^	1^**^	0.99^**^	−0.61	0.77	0.99^**^	0.99^**^	0.99^**^	1									
DTI (D)	0.99^**^	0.99^**^	0.99^**^	0.99^**^	0.99^**^	0.99^**^	0.99^**^	0.99^**^	0.99^**^	0.99^**^	−0.62	0.77	0.99^**^	0.99^**^	0.99^**^	0.99^**^	1								
DTI (S)	0.99^**^	0.99^**^	0.99^**^	0.99^**^	0.99^**^	0.99^**^	0.99^**^	0.99^**^	0.99^**^	0.99^**^	−0.62	0.77	0.99^**^	0.99^**^	0.99^**^	0.99^**^	1^**^	1							
STL DEN (D)	−0.98^*^	−0.97^*^	−0.96^*^	−0.96^*^	−0.96^*^	−0.96^*^	−0.96^*^	−0.95^*^	−0.96^*^	−0.94	0.64	−0.75	−0.98^*^	−0.97^*^	−0.97^*^	−0.96^*^	−0.96^*^	−0.96^*^	1						
STL DEN (S)	−0.98^*^	−0.97^*^	−0.96^*^	−0.96^*^	−0.96^*^	−0.96^*^	−0.96^*^	−0.95^*^	−0.96^*^	−0.94	0.64	−0.75	−0.98^*^	−0.97^*^	−0.97^*^	−0.96^*^	−0.96^*^	−0.96^*^	1^**^	1					
STL LEN (D)	0.98^*^	0.99^**^	0.99^**^	0.99^**^	0.99^**^	0.98^*^	0.99^**^	0.99^**^	0.99^**^	0.98^*^	−0.69	0.71	0.99^**^	0.99^**^	0.98^*^	0.99^**^	0.99^**^	0.99^**^	−0.96^*^	−0.96^*^	1				
STL LEN (S)	0.98^*^	0.99^**^	0.99^**^	0.99^**^	0.99^**^	0.98^*^	0.99^**^	0.99^**^	0.99^**^	0.98	−0.69	0.71	0.99^**^	0.99^**^	0.98^*^	0.99^**^	0.99^**^	0.99^**^	−0.96^*^	−0.96^*^	1^**^	1			
STL WID (D)	0.97^*^	0.98^*^	0.99^**^	0.99^**^	0.98^*^	0.98^*^	0.99^**^	0.99^**^	0.98^*^	0.97^*^	−0.69	0.68	0.98^*^	0.98^*^	0.97^*^	0.98^*^	0.99^**^	0.99^**^	−0.93	−0.93	0.99^**^	0.99^**^	1		
STL WID (S)	0.97^*^	0.98^*^	0.99^**^	0.99^**^	0.98^*^	0.98^*^	0.99^**^	0.99^**^	0.98^*^	0.97^*^	−0.69	0.68	0.98^*^	0.98^*^	0.97^*^	0.98^*^	0.99^**^	0.99^**^	−0.93	−0.93	0.99^**^	0.99^**^	0.99^**^	1^**^	1

* Correlation is significant at the 0.05 level.

** *Correlation is significant at the 0.01 level*.

### Transgenic plants exhibited improved oxidative stress tolerance and high proline accumulation

Drought and salt stress damage cells and changes in lipid composition and lipid peroxidation. Malondialdehyde (MDA), a naturally occurring product of lipid peroxidation and it is the important indicator for the oxidative damage of tissues. Therefore, to measure the drought and salt stress tolerance of transgenic plants we measured MDA content of transgenic and non-transgenic plants as an indicator of lipid peroxidation. Drought and salt stressed non-transgenic plants recorded greater MDA contents than transgenic plants. MDA contents increased with the duration of both the stresses (**Figure 9A**).

Electrolyte leakage indicates the cell membrane stability under abiotic stress conditions. A significance difference in electrolyte leakage was observed across the transgenic lines and non-transgenic (NT) plants under stress conditions. Electrolyte leakage was high (90%) in the NT plants and it is directly proportional to the severity of drought stress, while in the transgenic lines it was slightly less (60–70%) (**Figure 9B**). When compared to drought, salt stress caused more damage to the membrane resulting in considerable ion leakage in NT plants. Further, the electrolyte leakage increased in NT plants, with increase in severity of the salt stress. Whereas, the transgenic lines of *Ah-HDG11*-1, *Ah-HDG11*-2, and *Ah-HDG11*-6 maintained high membrane stability both under drought and salt stress.

**Figure 8:**
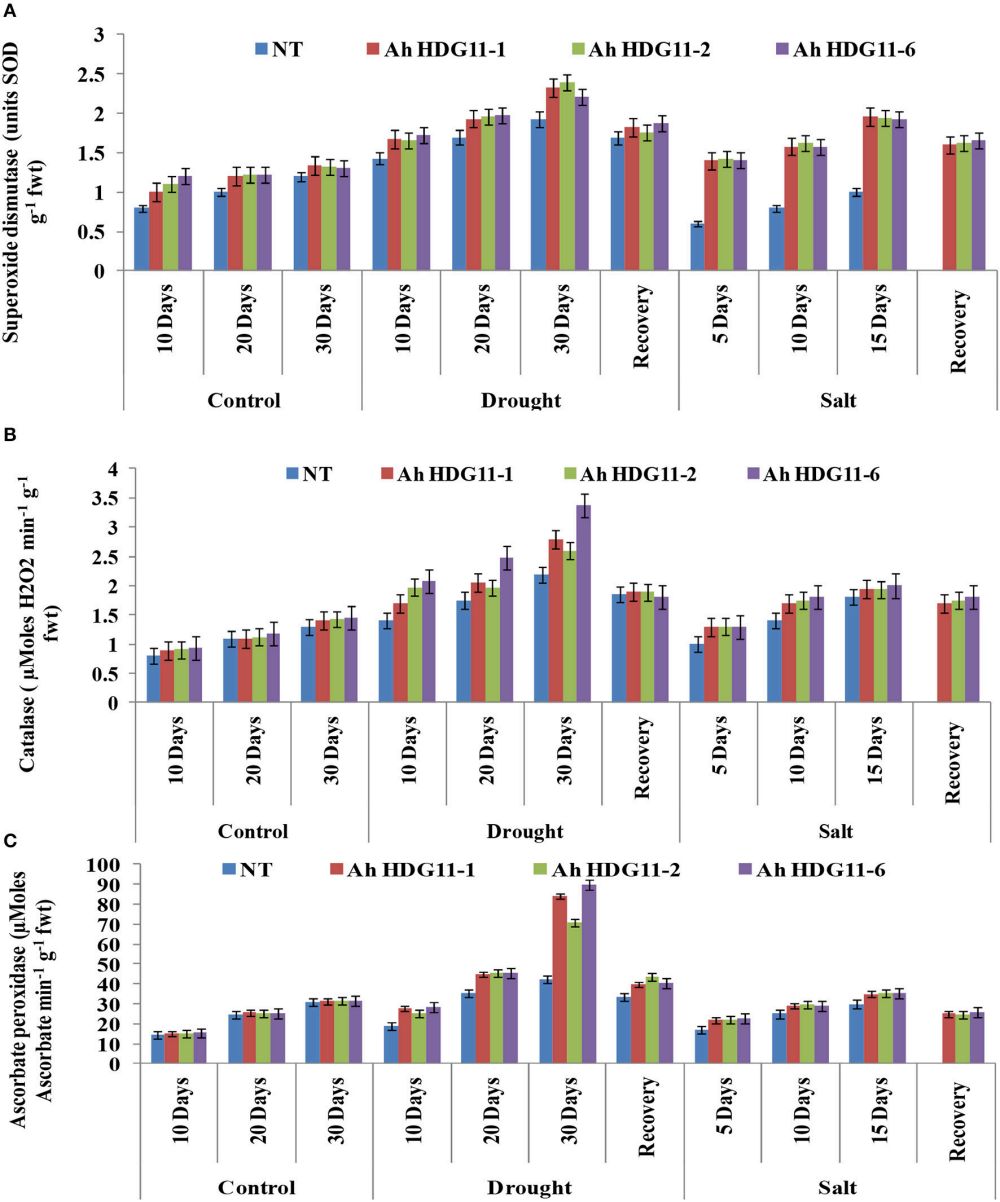
Physiological and biochemical analysis of transgenic lines *AhHDG11-1, AhHDG11-2, AhHDG11-6* along with the Non-transgenic (NT) plants. (**A)** Superoxide Dismutase (SOD) **(B)** Catalase (CAT) **(C)** Ascorbate Peroxidase (APX).

Further, to gain insights into the oxidative stress tolerance mechanism of transgenic plants, reactive oxygen species (ROS) scavenging activities were measured in both transgenic plants and the non-transgenic controls. Antioxidative enzymes SOD, CAT, and APX are the major enzymes responsible for scavenging ROS in plants under stress. A sharp increase in SOD, APX, and CAT activity were detected in transgenic lines of *Ah-AtHDG11*-1, *Ah-AtHDG11*-2, and *Ah-AtHDG11*-6 upon drought and salt stress and enzyme activities were found to be increased with the increase in severity of the stress (Figure 8). The heat map hierarchical clustering algorithm (Figure 11) clearly suggests that SOD, APX, and CAT enzyme activities under drought and salt stress condition were positively correlated with each other and negatively correlated with MDA and electrolyte leakage. Antioxidative enzyme activities have strong positive correlation with RL, chlorophyll content, and pod yield, RWC, TE, HI, DTI, STL LEN, and STL WID. However, SLA, STL DEN were negatively correlated with SOD, APX, and CAT enzyme activities under drought and salt stress conditions.

**Figure 9:**
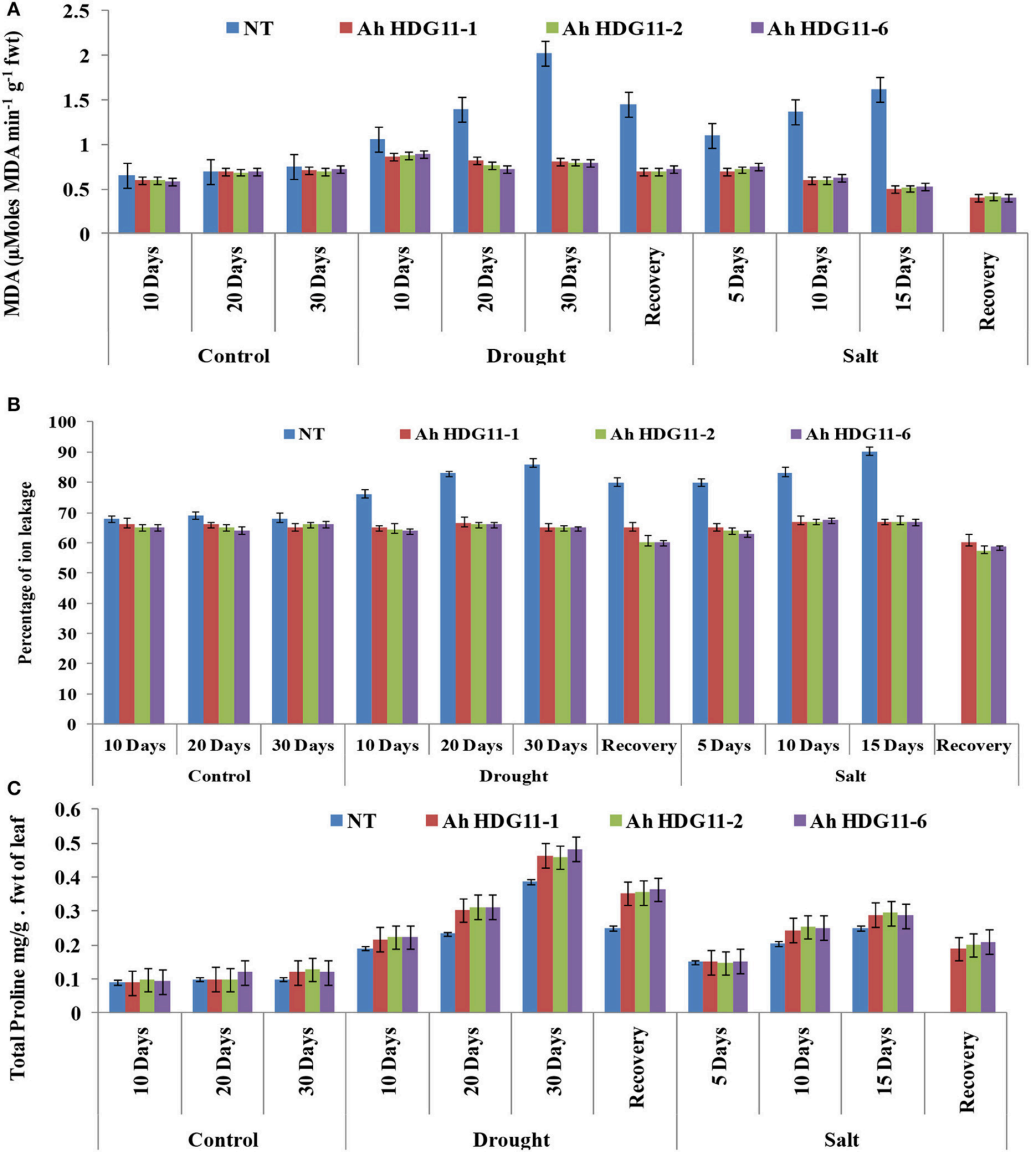
Biochemical analysis of transgenic lines *AhHDG11-1, AhHDG11-2, AhHDG11-6* along with the Non-transgenic (NT) plants. **(A)** Malondialdehyde (MDA) **(B)** Percentage of ions leakage **(C)** Proline (Pro).

A common phenomenon of abiotic stress response is accumulation of osmolyte proline. During drought and salt stress conditions, a three-fold increase in the proline was observed in the transgenic lines of *Ah-HDG11*-1, *Ah-HDG11*-2, and *Ah-HDG11*-6 compared to NT (Figure 9C).

### Elevated levels of known stress responsive genes observed in the transgenic plants

**Figure 10:**
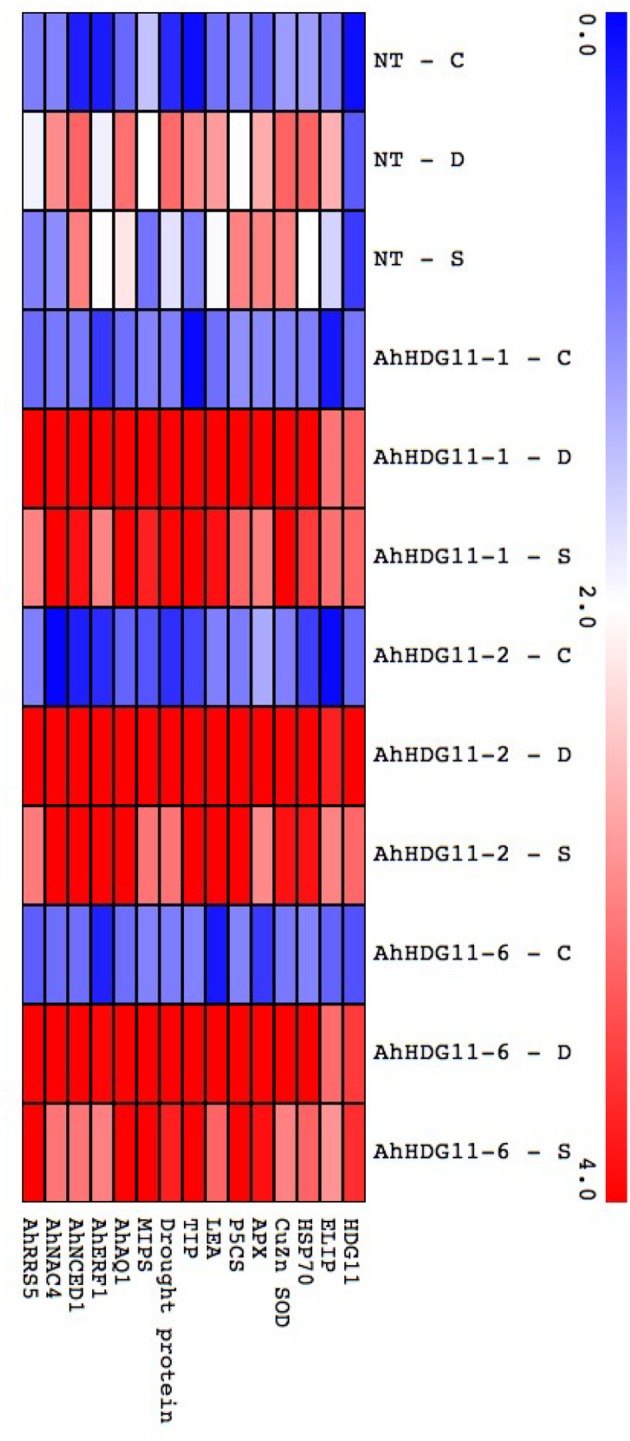
Gene expression analysis of known stress responsive genes in non-transgenic (NT) and Transgenic lines. qRT-PCR analysis of T_3_ generation peanut transgenic lines for the expression of known stress responsive genes under induced drought and salt lysimetric experiments. Heat map showing the expression of known stress responsive genes of stress gene network in non-transgenic (NT) and transgenic plants under well-water, drought and salt stress conditions. The log_2_ fold change scale is indicated in the heat map.

To figure out the mechanisms of drought and salt tolerance in transgenic plants, expression levels of known stress responsive genes in the stress gene network which codes for functional proteins *ELIP* (Early light induced proteins), *HSP 70* (heat shock protein 70), Cu/Zn SOD (superoxide dismutase), APX (ascorbate peroxidase), *P5CS* (Δ1-pyrroline-5-carboxylate synthetase), LEA (Late embryogenic abundant protein), TIP (Tonoplast intrinsic protein), Drought protein, *MIPS* (Major Intrinsic Proteins), *AhAq1* (Aquaporin), *AhNCED1* (9-cis-epoxycarotenoid dioxygenase1) and few of the stress responsive regulatory genes such as *AhERF1*(ethylene-responsive element binding factor), *AhNAC4* (NAM, ATAF, and CUC) family proteins 4), and *AhRRS5* (nucleotide binding site leucine rich repeat 5) were monitored using qRT-PCR. These stress responsive genes were selected based on their expression in earlier studies in *HDG11* transgenic plants i.e., *Cu/Zn SOD, APX, P5CS*, and *RRS5* whose expression levels were up-regulated in the transgenic plants (Yu et al., 2008). As revealed by the qRT-PCR data Figure 10, expression levels of known stress responsive genes were elevated in the transgenic and non-transgenic plants under drought and salt stress. However, the level of induction was more in the transgenic plants and expression levels of these stress responsive genes were correlated with the expression of *HDG11* transgenic in the transgenic plants. The obtained data clearly indicates all of these known stress responsive genes probably directly or indirectly regulated by the *AtHDG11* protein.

**Figure 11:**
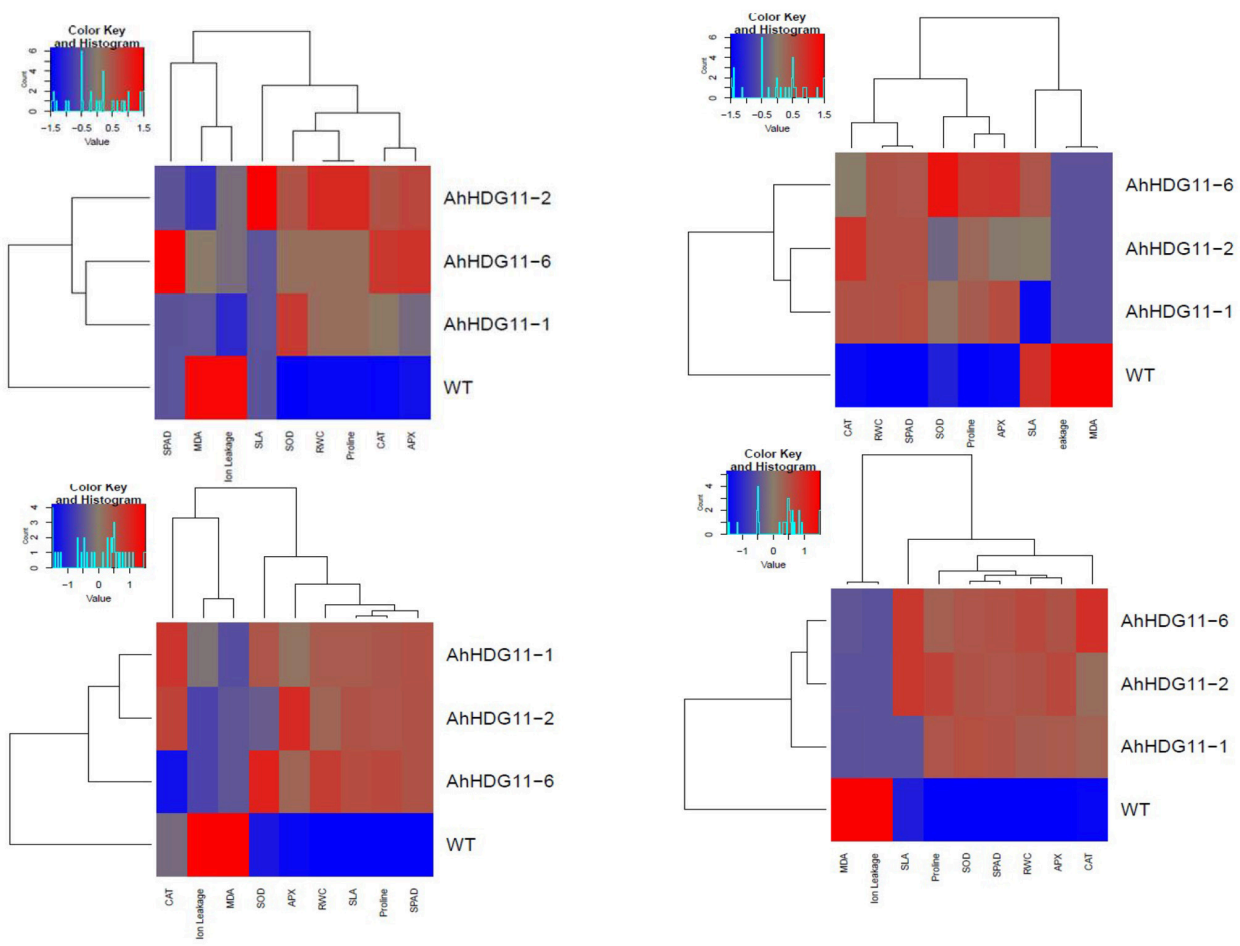
Overall assessments of physiological and biochemical responses in non-transgenic and transgenic lines during well-water **(A)**, drought stress **(B)**, re-watering **(C)**, and salt stress **(D)**. The relative values of physiological, biochemical parameters for the heat-map analysis. Scale: for the drought and salt tolerance capabilities, from brightest blue equal lowest to brightest red equal highest capabilities; for physiological and biochemical parameters, from brightest blue equals most decreased to brightest red equals most increased.

## Discussion

Soil moisture stress at various growth stages of peanut often limit the quality of production in SAT regions. A number of efforts were made to improve the traits such as cell turgor, positive carbon balance, cellular level tolerance and plant type for imparting stress tolerance and crop improvement in peanut under stress conditions (Rao et al., 2012). Lack of genetic and genomic resources has made molecular and traditional approaches faint in getting improved peanut varieties. One of the promising alternative ways is genetic manipulation. Since, these traits were controlled by several genes, transcription factors could be ideal candidates for genetic manipulation, toward crop improvement.

The present research describes stress inducible overexpression of *AtHDG11* in transgenic peanut plants that imparts drought and salt stress tolerance along with improved productivity. In earlier studies, *AtHDG11* transgene was expressed under constitutive promoter “*CaMV35S”*, hence, regardless of environmental cues plants produced extensive root systems and reduced stomatal density (Ruan et al., 2012; Yu et al., 2013, 2015; Li et al., 2016). Under favorable conditions when all the requirements are sufficiently supplied to the plant, larger root system is not required, and this plant type might pose metabolic load on yield potential (Blum, 2005). Therefore, in the present study *AtHDG11* gene expression was tightly regulated under a stress inducible promoter *rd29A*. The qRT-PCR data (Figure 1D) indicates that transgene expression was triggered by *rd29A* promoter under both drought and salt stress conditions.

A strong and efficient expression system is more advantageous for efficient gene functioning of desired traits in transgenic crops. Most of the studies that use constitutive promoter like *CaMV35S* cause adverse physiological effects and undesirable phenotypic characters in transgenic crops. Constitutive expression of transgene under unwanted conditions may also cause metabolic stress in transgenic plants. In recent past, several studies focused on stress inducible promoter of *rd29a, rab16a, Os ABA2, OS NCED3, BADH* etc. for achieving maximum stress tolerance in different transgenic crops (Shinozaki and Shinozaki, 1993; Zhang et al., 2008; Rai et al., 2009; Sun et al., 2010). Recently a maize *ZmGAPP* promoter (D8) was reported as suitable for alien gene expression under salt tolerance (Hou et al., 2016). Yi et al. (2010), reported, an abiotic stress inducible promoter *Wsi18* that functions in grain development of rice. In the present study transgenic peanut lines displayed several beneficial physio-morphological characters such as delayed wilting symptoms, better shoot and root ratio, improved biomass under drought and salt stress conditions. The resulted phenotypic data clearly indicates that stress regulated expression of *AtHDG11* is sufficient enough to obtain tolerant phenotype only under stress conditions, but not under ideal growth conditions. Analog phenotypes were observed in peanut transgenic carrying transgens under *rd29A* regulation (Kasuga et al., 2004; Bhatnagar-Mathur et al., 2007; Vadez et al., 2007; Devi et al., 2011; Jagana et al., 2012; Sarkar et al., 2014; Ramu et al., 2016).

Root architecture is an important agronomic trait that plays a major role in drought tolerance mechanism (Smith and De Smet, 2012) of crop plants. The greater water uptake capability of a plant depends on the greater development of lateral roots and deeper penetration ability of tap roots (De dorlodot et al., 2007; Comas et al., 2013). In this present study, transgenic peanut lines carrying *HDG11* exhibited extensive root system (50–60% more) with increased root nodules and biomass than NT. It has been established that *HDG11* directly binds to HD binding sites in the promoters of JA biosynthetic genes i.e., *AOS, AOC3, OPR3*, and *OPCL1* and increases the production of JA content in the root. The elevated levels of JA could further induce a cascade of auxin signaling genes thatpromote the lateral root formation besides up-regulating cell-wall-loosening proteins, that induce the primary root elongation (Xu et al., 2014; Cai et al., 2015). Deep and profuse root systems not only enhances drought tolerance, but also boosts the nutrient uptake and exclude the excess salt from the root cells through membrane transporters (Vadez et al., 2007). Thus, *HDG11* transgenic plants not only displayed drought tolerance but also showed salt tolerance.

Leaf water potential is a primary physiological index of plant water status and so considered the trait as an indicator to know the efficacy of the transgene. Higher water potential in the transgenic plants even under severe stress conditions could have possible by the extraction of water from the deeper layers of the soil due to profuse root growth and reduced transpiration associated with fewer stomata (Figure 6B). The elevated expression levels of three of the important aquaporin genes TIP (Regon et al., 2014), MIPS (Forrest and Bhave, 2007), and AhAq1 (Pan et al., 2009) in the transgenic plants in the present study could be the reason for the maintenance of high RWC in the leaves of transgenic plants. Plant aquaporins play an important role in maintaining the plant water relations (Maurel et al., 1993, 1997; Tyerman et al., 2002).

Stomata play a key role in gas exchange and water uptake with a strong impact on traits associated with transpiration, carbon assimilation, and WUE. The average stomatal density in *HDG11* transgenic peanut plants was reduced by 30% while stomata size increased by 20% compared with that of the NT plants under drought and salt stress conditions. The reduced stomatal density apparently contributed to the reduction in water loss that is evidenced by decreased transpiration rates in transgenic plants (Figure 6B). However, the reduced stomatal density does not affect the photosynthetic rates of transgenic plants (Figure 6A). The WUE which is a ratio of the rate of carbon assimilation to the rate of transpiration drastically increased in the transgenic plants (Figure 6C). Higher intrinsic instantaneous WUE data in the present study could be due to the low stomatal conductance and higher carboxilation capacity of the transgenic plants under drought and salt stress conditions (Gilbert et al., 2011).

Soil Plant Analytical Development (SPAD) chlorophyll meter readings (SCMR) and Specific leaf area (SLA) are easily measureable non-destructive surrogate traits of TE, used for selection of drought tolerant traits. Transgenic peanut lines recorded high SCMR and slightly reduced SLA under drought and salt stress conditions (Figures 7A,B). SCMR is an indication of the light-transmittance characteristics of the leaf which is dependent on the leaf chlorophyll content and density (Richardson et al., 2002; Arunyanark et al., 2008). Leaf chlorophyll content per unit area positively correlates with the photosynthetic activity of the leaf. Peanut transgenic lines with high SCMR under drought and salt stress could maintain a higher rate of photosynthesis per unit leaf area compared to non-transgenic plants. SLA indicates thickness of the leaves, and low SLA of transgenic lines in the present study specifies thicker leaves and more chlorophyll content per unit area. A similar phenotype of thick leaves with tightly packed mesophyll cell and higher photosynthesis rate under drought stress has been reported for the *Arabidopsis ERECTA* gene, a putative leucine-rich-repeat receptor protein kinase gene (Masle et al., 2005). In the present study the expression of a nucleotide binding site leucine rich repeat 5 (*AhRRS5*) which is a homolog of *ERECTA* showed enhanced expression in the transgenic plants under drought and salt stress condition (Figure 10). As reported earlier, *Arabidopsis ERECTA* gene was trans activated by *HDG11* protein (Yu et al., 2008). Thus, in this study, the increase in the leaf thickness in the transgenics might be associated with the up regulation of *ERECTA* like genes. The increase in shoot biomass and reduction in SLA supports the possibility for greater assimilation in the transgenic lines under drought and salt stress. Previous studies on peanut suggest that SLA and SCMR correlates with WUE. As reported earlier in peanut (Rao et al., 2001; Sheshshayee et al., 2006; Songsri et al., 2009) a strong positive correlation between WUE and SCMR, and a negative correlation between SLA and SCMR have been observed in the present study. Therefore, high SCMR and low SLA of *HDG11* transgenic lines specify the greater WUE of transgenic peanut lines compared to non-transgenic plants.

One of the well-studied compatible solute is the amino acid proline, which is a multi-functional molecule that plays a major role in plant abiotic stress defense. It is actively involved in osmoregulation, scavenging of free radicals, and as a molecular chaperone for stabilizing protein structure, thus protects plant cells from the damaging effects of various environmental stresses (Parvaiz and Satyawati, 2008; Verbruggen and Hermans, 2008; Szabados and Savouré, 2010; Koyro et al., 2012; De Carvalho et al., 2013). A high level of proline content (30–40%) and elevated levels of *P5CS* gene expression was recorded in peanut transgenic lines compared to NT both under drought and salt stresses (Figures 9C, 10). Transgenic peanut plants not only accumulated high solutes, but also exhibited higher membrane integrity under severe stress conditions. Transgenic peanut lines overexpressing *HDG11* showed significantly reduced electrolyte leakage and MDA levels (Figure 9A), thus display greater membrane stability (Figure 9B) compared to NT under drought and salt stress conditions. In totality, the obtained data clearly indicates that membrane is better protected from stress-induced ROS toxicity. One of the most important defense mechanisms against abiotic stress is the antioxidant system, which detoxifies ROS molecules such as hydrogen peroxide, superoxide, and singlet oxygen and keeps an adequate cellular redox balance (Noctor and Foyer, 1998; Asada, 1999). Supporting this, higher levels of antioxidative enzymes such as SOD, APX, and CAT were recorded in the *HDG11*-overexpressing peanutplants (Figure 8). Further, the antioxidative enzyme activities were in consonance with APX and Cu/Zn SOD gene expression levels in the transgenic plants (Figure 10), therefore protecting the plants from oxidative damage by enhancing ROS scavenging capability. The results obtained in the present investigation were in agreement with the altered antioxidative system in the transgenic tobacco (Yu et al., 2008), rice (Yu et al., 2013), tall fescue (Cao et al., 2009), sweet potato (Ruan et al., 2012), cotton and poplar (Yu et al., 2015), and wheat (Li et al., 2016).

In the present study we observed that shoot and root lengths, biomass were significantly (30–40%) higher in transgenic lines compared to non-transgenic plants (Figures 4A–C,F, 2A–C). Transgenic peanut lines also displayed greater pod number, pod, and seed weight, HI compared to NT under drought and salt stress conditions (Figures 3A–D). Present data of peanut transgenic are in consistent with the data presented in a previous study of transgenic rice over expressing *HDG11* that resulted in greater yield under drought stress conditions. A well-equipped WUE traits and fine-tuned defense mechanisms might be contributing to the enhanced biomass and yield in the transgenic peanut plants under drought and salt conditions (Asif et al., 2011; Qin et al., 2011; Yu et al., 2013; Bhauso et al., 2014; Manjulatha et al., 2014; Pruthvi et al., 2014).

Present study has successfully demonstrated the stable integration and stress inducible overexpression of *AtHDG11* in peanut with improved drought and salt tolerance. Transgenic plants displayed impressed biochemical and physiological traits like (1) Efficient water extraction from deeper layers of soil demonstrated by enhanced root lengths and later roots; (2) Minimized water loss through the stomata evidenced by reduced stomatal density; (3) Enhanced WUE traits, elevated photosynthetic rates to minimize the yield loss under stress conditions; (4) Cellular stress tolerance with increased accumulation proline, LEA, ELIPs, and efficient ROS detoxification mechanism.

## Author contributions

JB, VP, SK, and KG: Performed the experiments; TC, CA, SP, and CP: Analyzed the data; CP: Conceived and designed the experiments; JB, TC, and CP: Wrote the paper.

### Conflict of interest statement

The authors declare that the research was conducted in the absence of any commercial or financial relationships that could be construed as a potential conflict of interest.
