# Supplementary material for: Stress Inducible Overexpression of AtHDG11 Leads to Improved Drought and Salt Stress Tolerance in Peanut (Arachis hypogaea L.)
Source: Front Chem. 2018 Mar 2;6:34. doi: 10.3389/fchem.2018.00034 (PMC5840212; doi:10.3389/fchem.2018.00034)
Supplement: Supplementary file 1 [file Table1.DOC]

| **S.NO** | **Name of the gene and Accession number** | **Forward Primer**  **5’ 3’** | **Reverse Primer**  **5’ 3’** |
| --- | --- | --- | --- |
| 1 | ELIP and AF479309 | ATGGAAGGCTTGCAATGATTG | CCACACTAGTCCCCAAGAACCA |
| 2 | Thioredoxin JR555818 | CACCCAATGGTGTGGTCCTT | TTTGCCAATGGCTTGTTCTCT |
| 3 | HSP70 EZ733089 | AAGGACATAAGTGGCAACCCTAGA | TCCCTCATACAGCGAATCAATTT |
| 4 | CuZn SOD EZ722218 | GAAATGGTCCAACCACTGTGACT | TGAAATGCGGTCCAGTTGAC |
| 5 | APX  KC594039.1 | CCCCTCATCTTTGACAACTCT-3’ | GCATCTTCATCCGCAGCATA |
| 6 | P5CS | GTCCTGTAGGAGTTGAGGGTTTG | TTTAGTGGCAGTTCTTTATGAGTGT |
| 7 | nsLTP FS980595 | GATGAAGAAGGTGTGTGCAGTGTT | GTGATGGCCCCAAGACATG |
| 8 | LEA HM543589 | ATCACTTCAAACAAAACGAGCTTTAA | TTGTGCTGTTGTCGCATATCC |
| 9 | TIP EZ751999 | TAGCTGCTGGCCCATTCAGT | CAAGCCCCCACCAATCAAC |
| 10 | Drought protein ES753436 | TCACGAAATCCATGGAACCA | TTTCATAAAGAACCTCCGCATTC |
| 11 | MIPS ES752304 | CGCTCCAAGGAAATCTCCAA | TCCCCCACATATGGCACATA |
| 12 | AhAQ1  JQ048930 | 5’-ACCCTCCTCTTC CTCTACATC-3’ | 5’-CAAGCACTGAGCCA CCATAT-3’ |
| 13 | ahERF1 | GGCAGAGGCCCTGGGGCAAG | GCCGAGCCAGACACGGACCC |
| 14 | AhNCED1 | TTACCTGTGGGATTGTTTGC | ACATGAGCCTCTACTTCTGC |
| 15 | AhNAC4 | GCCAGAGATCGACGACCGTT | GGCTACGAGACCCTGTTCCTG |
| 16 | AhRRS5 | GCAGTGATGAACGCAGCACTCTCTC | GTGCTGACATCTGTACAGGAAGTGAG |
| 18 | Actin EZ723877 | CACATGCCATCCTTCGATTG | CCAAGGCAACATATGCAAGCT |

Supplementary Table 1. List of qRT-PCR primers used for the study.
