# Supplementary material for: Stress Inducible Overexpression of AtHDG11 Leads to Improved Drought and Salt Stress Tolerance in Peanut (Arachis hypogaea L.)
Source: Front Chem. 2018 Mar 2;6:34. doi: 10.3389/fchem.2018.00034 (PMC5840212; doi:10.3389/fchem.2018.00034)
Supplement: Supplementary file 2 [file DataSheet1.DOC]

**Supplementary Figure 1. a.** Schematic diagram of the T-DNA region of the pGreen0029*:Atrd29A::AtHDG11:Poly A* expression vector. **b.** Various stages of *Agrobacterium tumefaciens*-mediated genetic transformation of peanut var. JL-24.

**Supplementary Figure 2.a.** Initial screening of T_1_ putative transgenic seeds on Kanamycin media to identify the transgenic plants. **b.** PCR analysis of T_1_ generation plants for *AtHDG11* transgenic plants using *nptII*, *rd29A* promoter and promoter-*HDG11*gene gene specific primers (M: Marker, P: Positive control, NT: Non transgenic B: Negative control). **c.** PCR analysis of T_2_ generation plants. **d.** Genomic southern analysis of the T_2_ generation peanut transgenic plants for the integration and copy number of the transgene (P: binary vector; NT: Non transgenic; B: Mock plants; 1-7 transgenic peanut plants.


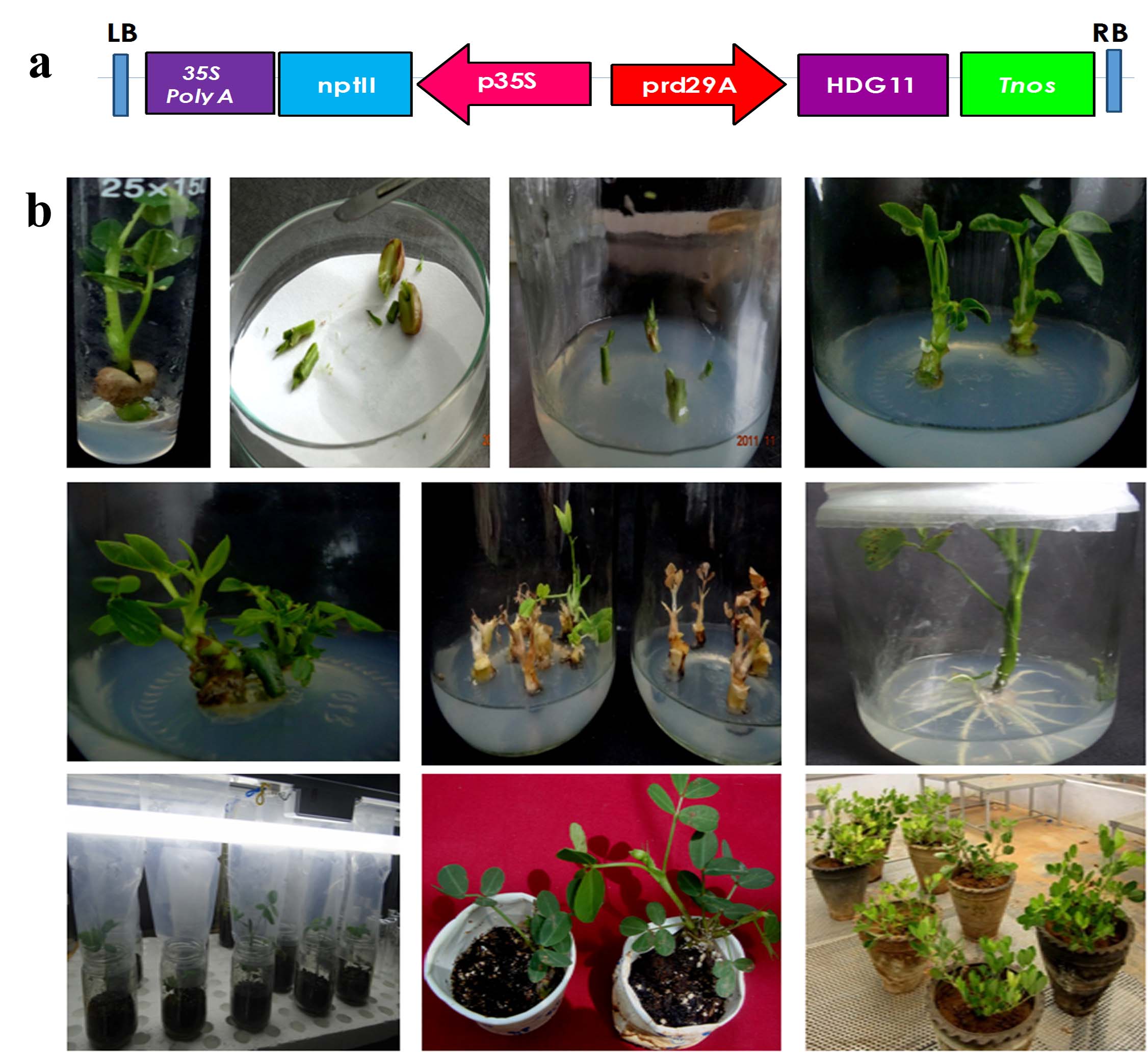


**SupplementaryFigure 1.**

**
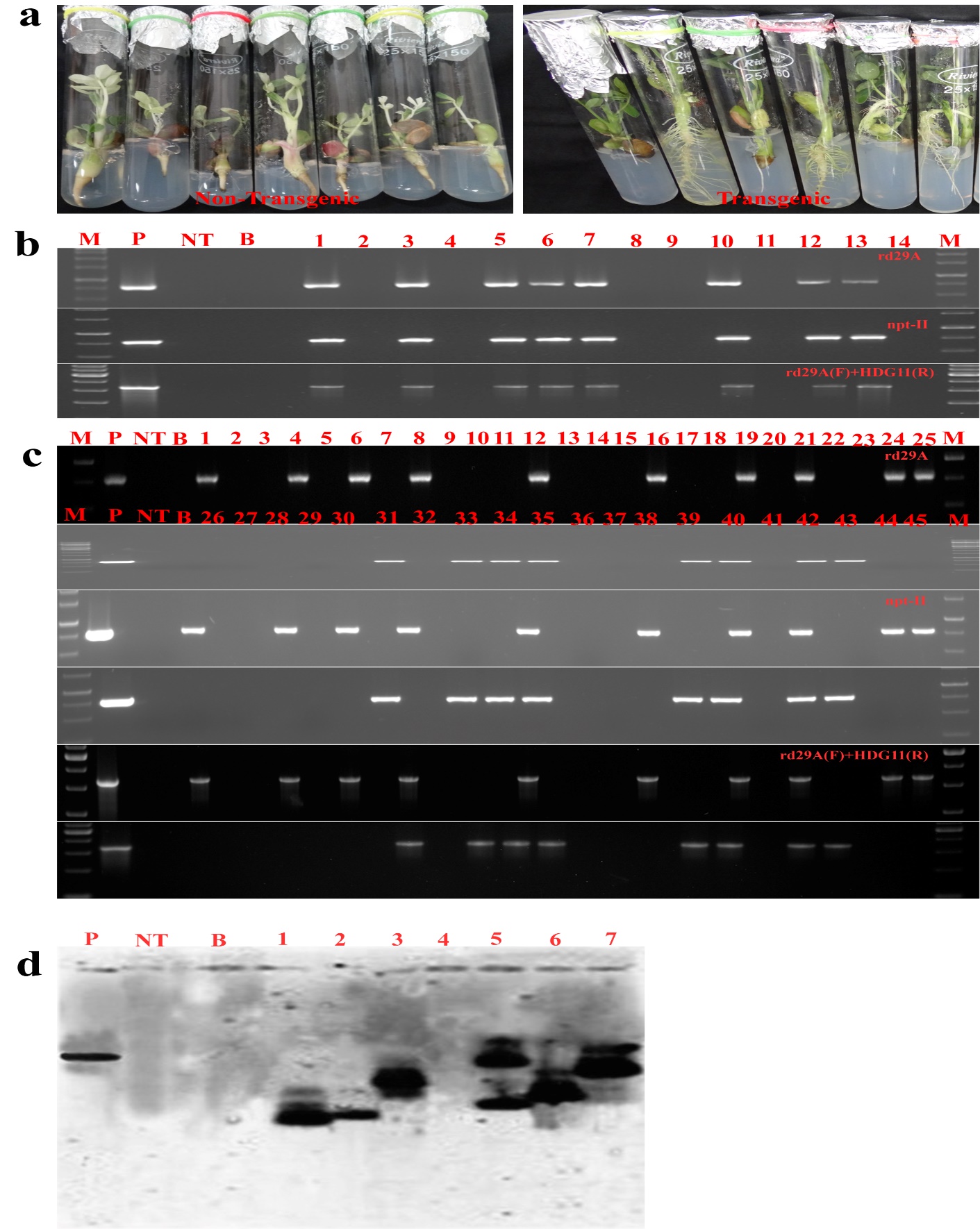
**

**Supplementary Figure 2.**
